# Simultaneous Enhancement of the Mechanical Properties, Performance and Insensitivity of an Energetic Elastomeric Polyurethane Binder by Kinetically Grafting Reactive Spiranes

**DOI:** 10.3390/polym15234564

**Published:** 2023-11-28

**Authors:** Mingyang Ma, Younghwan Kwon

**Affiliations:** 1 School of Chemistry and Materials Science, East China University of Technology, Nanchang 330013, China; m_ma@ecut.edu.cn; 2 Department of Chemical Engineering, Daegu University, Gyeongsan 712-714, Republic of Korea

**Keywords:** polyurethane, reactive plasticizer, click reaction, binder, ring strain energy

## Abstract

A series of robust energetic polyurethane binders was developed by in situ grafting reactive spiranes to achieve the migration-resistant processing aid and compensate for the energy output. The reactive grafting spiranes (RGSs), bearing two highly ring-strained spiranes, were synthesized sequentially to provide a promising ring strain energy up to a maximum value of 290 kJ mol^−1^. The thermodynamic compatibility of the RGS with uncured glycidyl azido polymer (GAP) was studied quantitatively by analyzing the glass transition temperature of their blendings. The reactivity study of the catalyst-free click reaction with respect to spacer-dependent species was amplified by tracing the extent of the reaction and measuring the activation energy. The faster reactivity of propargyl species was evident from two experimental approaches, which were verified further by theoretical predictions. Interestingly, the energy gap difference in the frontier molecular orbitals agreed well with the difference in activation energy between the two types of spacer-dependent species. The mechanical and thermochemical enhancements of GAP-based polyurethane with RGS were basically gained from those highly ring-strained moieties.

## 1. Introduction

As a class of versatile polymer, polyurethanes (PUs), have been widely used in a variety of fields for several decades since the first polyurethane was synthesized by Dr. O. Bayer in 1937 [1]. Polyol and isocyanate, regarded as the soft and hard segments, respectively, are two basic building blocks for the preparation of PUs. According to the diverse combinations of polyols and isocyanates in terms of structures, contents and fabrication methods, the PU-based materials can suffice for applications from elastic soft matters to hard rigid plastics. PU elastomers with superior mechanical characteristics find applications as polymer binders in plastic-bonded explosives (PBXs). PBXs are energetic composites composed of highly energetic materials bound together by polymer binders that can produce a void-free and protective matrix [2]. The intense demand for the next-generation PBXs with ideal properties, such as low sensitivity, high energy density, improved mechanical integrity, etc., has triggered the development of formulated ingredients [3]. As a processing aid in the manufacture of PBXs, energetic plasticizers are generally added to ease the processibility of composite formulations, offer improved performance and tune the mechanical properties of cured polymer composites [4,5,6]. One of the focal points regarding safety is the fact that energetic plasticizers are prone to migrate from the polymer matrix, which inevitably destroys the mechanical integrity of the PBX, leading to susceptibility to accidental detonation [7]. Physical interactions (i.e., intermolecular force) between polymer and plasticizer are unstable under mechanical or thermal stimuli for long-term use. A rationalized approach to attaining the zero migration of plasticizers has been studied extensively. The novel strategies solving the migration problems of energetic plasticizers in the glycidyl azido polymer (GAP) were developed using a series of reactive energetic plasticizers (REPs), which permanently became an integral part of GAP-based polyurethane (PU) binders [8,9,10,11,12,13,14].

The safe handling, transport and use of energetic ingredients are critical to safety issues, because most energetic ingredients are functionalized by sensitive explosophore groups, such as -ONO_2_, -N_3_ or -NO_2_ groups [15,16,17,18]. Although those conventional explosophore groups have disadvantages, such as high sensitivity, being detrimental to the environment and easy oxidation in air, the replacement of those groups is still a challenging task considering the need for both energetic performance and safety [18,19]. Ring strain energy (RSE) has emerged as a benign, robust and stable energy source that has attracted considerable interest because the liberation of ring strain gives rise to a more appreciable increase in energy output and more compact structures than acyclic species [20]. Importantly, the release of RSE is basically dependent on a typical ignition process rather than external stimuli. One of the traditional approaches utilizing the highly ring-strained hydrocarbon is associated with the ring-strained polymer binder for a solid propellant, such as benzvalene, norbornadiene or cubane [21,22]. On the other hand, the labile features of those polymer binders, e.g., susceptibility to severe mechanical stretching, have limited their further use as binders. The mechanical instability and low elasticity might be caused by the anomalous bond angles and lengths of ring-strained hydrocarbons, which were constructed as the backbone unit. Ring-strained hydrocarbons attracted interest when they were developed as a scaffold for high-energy explosives, such as octanitrocubane, CL-20, etc., [23,24].

Spiro[3.3]heptane (SH) and dispiro[3.1.3^6^.1^4^]decane (DSD), which have two and three cyclobutanes joined perpendicularly or near-perpendicularly by a common tetracoordinate carbon atom, can provide RSEs of ca. 200 and 300 kJ mol^−1^, respectively, based on empirical theory [20,25]. The unique structural features of spirane compounds are of great interest, and explicit studies of their configurations, physical properties, reactivities, and strain energies have been performed [26,27,28,29,30,31,32]. In particular, Koch and Wineman patented dispirane hydrocarbons as high-energy rocket fuels [33,34]. In this work, reactive grafting spirane (RGS) compounds, which replaced the explosophore groups with SH and DSD as the ring-strained building blocks, were synthesized sequentially starting from cyclobutane derivatives. In addition, the effects of electron-withdrawing groups (EWGs) adjoining the alkyne group on click reactivity were examined in detail. The computational predictions of the frontier molecular orbitals (FMOs) corresponding to the spacer-dependent species were correlated successfully with the measured activation energy. The effects of ring-strained building blocks on the thermal, mechanical and thermochemical characteristics, as well as the migration resistance of RGS@GAP-based binders, were investigated.

## 2. Results and Discussion

### 2.1. Synthesis of RGS

Buchta et al. reported the synthetic route to SH and DSD moieties [35]. A modified version of the procedure reported by Buchta was used to synthesize the precursor for RGS. Diethyl cyclobutane-1,1-dicarboxylate, as the initial reagent, was reduced to the diol by LiAlH_4_, which was then converted to bis-*p*-toluenesulfonate. The latter gave rise to cyclization in the presence of a diethyl malonate sodium salt via condensation to form the SH diester. The SH diester is of great importance because it is used not only as the building block for the synthesis of 2-propynyl spiro[3.3]heptane-2-carboxylate (PSHC, *n* = 1) and 3-butynyl spiro[3.3]heptane-2-carboxylate (BSHC, *n* = 2) but also as the starting material destined for 2-propynyl dispiro[3.1.3^6^.1^4^]decane-2-carboxylate (PDSDC, *n* = 1) and 3-butynyl dispiro[3.1.3^6^.1^4^]decane-2-carboxylate (BDSDC, *n* = 2), where *n* represents methylene spacers between ester and alkynyl. Basically, the *gem*-diacid is undesirable because of the high possibility of forming highly crosslinked networks that may limit their application as elastomers with a high elasticity. To obtain the mono-functional acid with respect to the SH-based RGS, the diester was hydrolyzed primarily using a KOH/EtOH aqueous solution, followed by adjusting the pH of the solution to 3. The *gem*-diacid was then decarboxylated in pyridine to form a mono-functional acid. Finally, PSHC and BSHC were synthesized through an esterification reaction between SH acid and alkynols. The synthetic route to PDSDC and BDSDC was the same as that of the SH-based RGS but with a slightly lower yield. From the first step to the end step, the overall yields of spiro- and dispiro-RGS were about 52% and 41%, respectively.

### 2.2. Prediction of Ring Strain Energy

**Figure 1:**
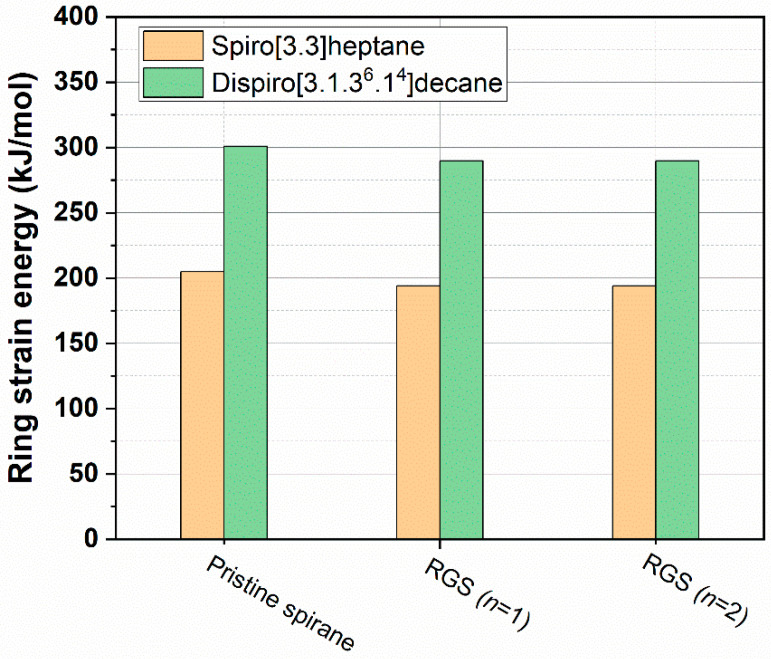
Computed ring strain energy of the pristine spirane and RGS.

A reasonable explanation of RSE is depicted appropriately by the extra energy manifesting as a significant increase in the heat of formation (Δ*_f_ H^o^*) compared with its strain-free isomer [20]. The substituents attached adjacent to the cyclics, as distinct from the intact cyclic compound, will definitely affect the electron density distribution and alter the pristine RSE to some extent. To predict the theoretical RSE in RGS, which facilitated an understanding of its specific energetic contribution, homodesmotic schemes were considered, primarily owing to the successful prediction for the diverse RSEs of the cyclics [36,37]. Two types of homodesmotic schemes were provided in Equations (S1) and (S2) (see Supporting Information). Figure 1 shows the computed RSE for the pristine spirane and RGS. As expected, the RSE of the pristine DSD was 301 kJ mol^−1^, which was 96 kJ mol^−1^ higher than the pristine SH. This difference is in good agreement with the predicted results of approximately 100 kJ mol^−1^ of 1,1-dimethylcyclobutane [36,38]. On the other hand, the predicted RSE of RGS decreased with an average of approximately 10 kJ mol^−1^ for the two building blocks independent of *n*. The observed reduction in RSE could be attributed to stabilization by the neighboring substituents [36,39,40]. A similar decrease in the propargyl and 3-butynyl species suggested that the extent of the stabilization of cyclics appeared to be mainly dependent on the neighboring substituents.

### 2.3. Processing Performance of RGS

**Figure 2:**
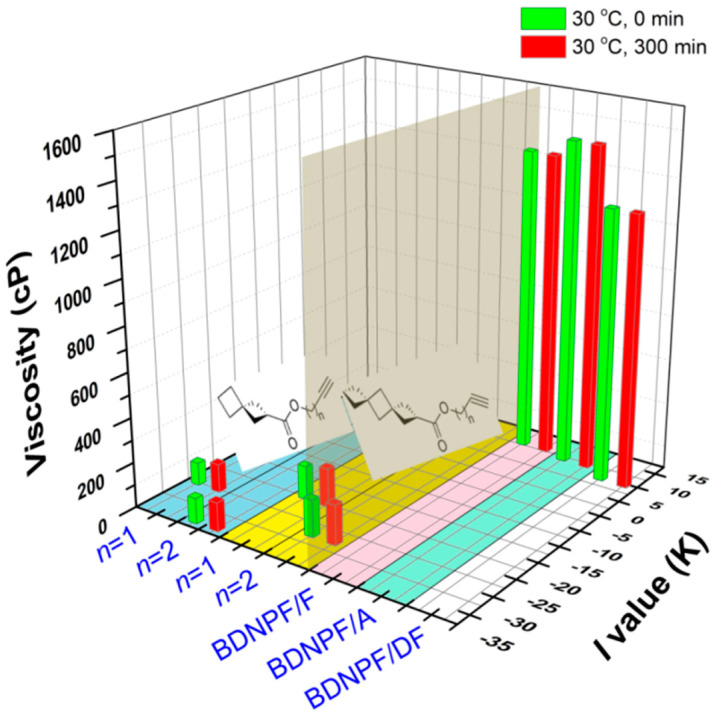
Comparison of the processing effect between RGS and energetic plasticizers in terms of the *I* value and viscosity. Compared with three types of commercially available *gem*−dinitro energetic plasticizers, the RGS showed a superior processing effect on the uncured GAP.

The thermodynamic compatibility of the polymer and processing aid is demonstrated ostensibly as a homogeneous mixture that shows a single *T*_g_. The composition-dependent *T*_g_ of the RGS and GAP prepolymer binary mixtures with different compositions between GAP and RGS ranging from 80/20 *w*/*w* to 50/50 *w*/*w* was measured via DSC (see Supporting Information Figure S9). Figure S10 presents the summarized *T*_g_ of the binary mixtures with an inclusion of RGS at three weight fractions. In accordance with the thermodynamic compatibility, a single *T*_g_ appeared after the temperature regime, ranging from −120 to 0 °C. The *T*_g_s of the binary mixtures decreased with increasing RGS content in the GAP prepolymer, suggesting their mutual compatibility. In addition, the extent of the shifted *T*_g_ appears to be pertinent from the content and structural characteristics of RGS. The structure-dependent effects of RGS on the compatibility with uncured GAP were approached quantitatively using Equation (S3) (see Supporting Information) [41]. *I* is the interaction parameter, which represents the ability to decrease the *T*_g_ of a polymer. The RGS, having higher processing capability, usually provided lower *I* values, as calculated by Equation (S3). Figure 2 shows the calculated *I* values of PSHC (−25.0 ± 0.4 K), BSHC (−33.4 ± 0.5 K), PDSDC (−20.1 ± 0.3 K) and BDSDC (−27.7 ± 0.6 K), respectively. An observational increase in the *I* value from *n* = 2 to *n* = 1 expressed a depression in the flexibility of the GAP/RGS mixtures. This phenomenon was also observed in a previous study [11]. One explanation for this is that the increased chain length from *n* = 1 to *n* = 2 results in high free volume [39,42,43]. The degree of compatibility between RGS and uncured GAP in terms of the configuration of the building blocks can be ranked as SH > DSD. This was attributed to the steric clashes originating from the concomitant increase in the spirocyclic size of the RGS. The lower flexibility of the bulky substituent may hinder the molecular motions of the RGS while weakening the interactions between RGS and uncured GAP, eventually causing a higher *T*_g_ in the resulting blendings. As reported previously [11], the *I* values of several commercially available energetic plasticizers (EPs) were compared with those of RGS. As shown in Figure 2, three types of EPs exhibited inferior compatibility with the uncured GAP in contrast to RGS.

In the fabrication of energetic PU binders, the addition of a miscible low-viscosity processing aid can offer better processability to the formulated compounds. The viscosity of the GAP/RGS binary mixture was evaluated due to a processing concern about whether RGS reacts quickly with the GAP prepolymer, i.e., forming grafted RGS. The highly grafted RGS, which gives rise to more complicated entanglement and resistance to motion in GAP molecules, will definitely lead to an increase in the viscosity of the GAP prepolymer. The time-dependent viscosity variation of the uncured GAP/RGS (1/1, *w*/*w*) blending was monitored at room temperature, aiming to maintain the same experimental conditions for fabricating the PU binders. Figure S11 (Supporting Information) illustrates an isothermal plot for RGS and uncured GAP mixtures. The viscosity of the GAP prepolymer was measured to be 6000 cP at processing temperature and decreased to 100–170 cP after adding 50 wt% of RGS, indicating the excellent processing effect of RGS. As shown in Figure 2, the viscosities of all binary blendings were maintained at approximately 130–180 cP after consecutive measurements for 300 min, suggesting that the RGS would not be of concern regarding the troublesome issue of proneness to an increase in the viscosity of the formulations due to the click reaction. The viscosities of the binary mixtures were much lower than those of the *gem*-dinitro energetic plasticizers/GAP prepolymer mixtures, indicating that the RGS could allow a higher solid loading.

### 2.4. Catalyst-Free Click Reactivity

The processibility of GAP-based binders via the inclusion of RGS was closely associated with their click reactivities. An explicit understanding of the relative click reactivity of RGS toward GAP is in favor of customizing a proper processibility for a specific formulation. The bulk click reaction between RGS and uncured GAP was implemented at a theoretical azide/alkyne stoichiometric ratio of 1/0.5 under 60 °C. A series of azide–alkyne click reactivities between RGS and uncured GAP were investigated by tracing the reaction extent using a ^1^H NMR spectrometer. Figures S12 and S13 present the comparative ^1^H NMR spectra during the click reaction of RGS with uncured GAP. For instance, the two methylene protons of spirane adjacent to a carbonyl group of SH-based RGS, which are denoted as **1**, were selected as the reference to trace the click reaction because of their lesser effect on the chemical shift after forming the isomers. The proton variations of the –CH_2_ adjacent to the –COO– of PSHC and BSHC, marked as **2** and **4**, respectively, showed that the progress of click chemistry was detectable. The signals of peak **2** and **4** were continuously depressed and fully depleted after approximately 72 and 100 h (see Supporting Information Figure S12). Those newly generated peaks attributed to 1,4- and 1,5-triazole regioisomers were found to shift downfield and are marked as **3**, **3′**, **5**, **5′**, respectively. The conversion of four types of RGS was then plotted against the corresponding reaction time (see Supporting Information Figure S14). The –N_3_ group, which is an ambiphilic dipole, would yield 1,4- and 1,5-triazole regioisomers when reacting with an alkyne (dipolarophile) induced by a purely thermal approach. The similarity between the energy gaps of the interactions from HOMO_dipole_ to LUMO_dipolarophile_ and from HOMO_dipolarophile_ to LUMO_dipole_ leads to the formation of isomers [44]. As expected, the catalyst-free click chemistry of the RGS and uncured GAP was completed within 7 days, concurrently matching the curing time of a PU reaction. The RGS (*n* = 1) facilitated the click reaction better than RGS (*n* = 2). This showed a high degree of agreement with a previous study that showed a significant decrease in the LUMO levels of dipolarophiles affected by different adjacent EWGs [11]. Basically, RGS with *n* = 2 had fewer electron-deficient alkynes than those with one spacer (*n* = 1) because of the inductive effect.

**Figure 3:**
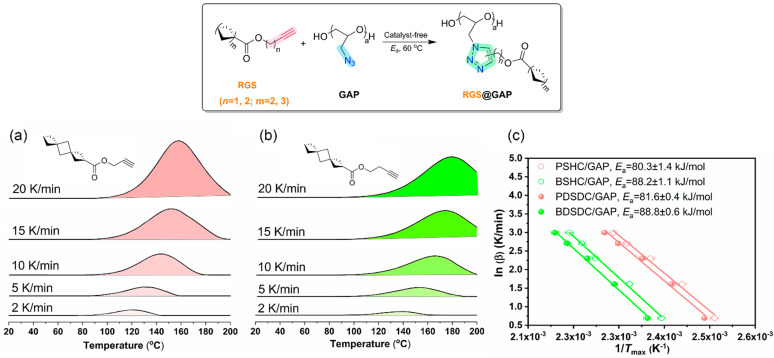
Dynamic DSC thermograms of PDSDC/uncured GAP (**a**) and BDSDC/uncured GAP blendings (**b**) (exothermal, up). (**c**) Linear Arrhenius plot of ln(*β*) against $T_{\max}^{-1}$.

The activation energy (*E*_a_) of Huisgen-type cycloaddition was measured via DSC due to its inherent exothermal behavior. The pathway to obtaining the *E*_a_ is based on variable heating rates and typically include at least three experiments ranging from 1 to 20 °C min^−1^. The *E*_a_s values were then calculated according to Equation (S4) (see Supporting Information) [45,46,47]. Figure 3 presents two sets of DSC curves concerning the uncured GAP with (a) PDSDC and (b) BDSDC at five heating rates (for another series see Supporting Information Figure S15). All *T*_max_s shift to high temperature intervals when the heating rates were increased. As the same heating rate was compared, the observed lower *T*_max_ of the propargyl-based RGS in contrast to the 3-butynyl-based ones indicated faster click reactivity. As shown in Figure 3c, the *E*_a_s values of 3-butynyl-based RGS were distributed between 88.2 and 88.8 kJ mol^−1^ and were greater than these of propargyl-based ones, which ranged from 80.3 to 81.6 kJ mol^−1^. This is in concert with the aforementioned kinetics study. As expected, the determinant for the click reaction of the RGS was attributed primarily to the number of CH_2_ moieties and independent of the structures of the building blocks.

**Figure 4:**
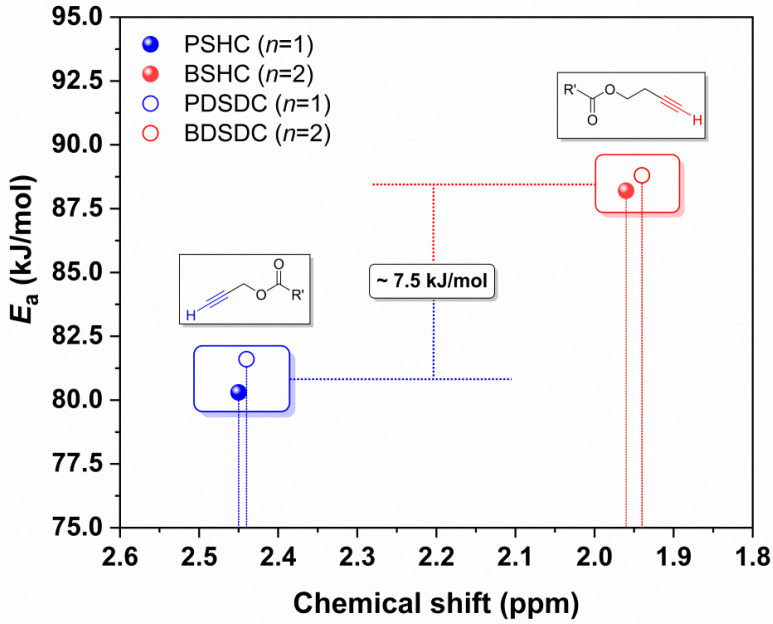
Relationship between *E*_a_ and the deshielding degree of alkyne groups. *R*’ groups represent the spirane building blocks.

The difference in *E*_a_ with regard to the spacer-dependent species (i.e., *n* = 1 vs. *n* = 2) was related to the deshielding degree of the alkyne group of RGS. The *E*_a_ value of 3-butynyl-based RGS containing the chemical shift of a terminal proton of alkynyl ranging from 1.94 to 1.96 ppm was higher than that of propargyl-based RGS in the vicinity of 2.45 ppm (Figure 4). Accordingly, the observation of the upfield to downfield shift was indicative of a more electron-deficient alkyne of propargyl species, leading to a lower *E*_a_.

**Figure 5:**
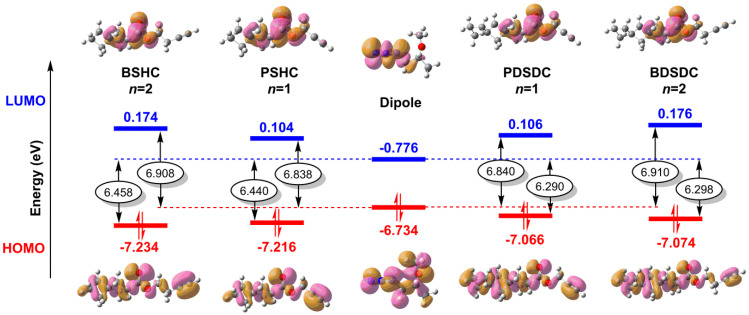
FMO energy level diagram of the dipole (azido model) and dipolarophile (RGS) computed at B3LYP/6−31G*.

A theoretical explanation of how a neighboring EWG accelerates the click reaction requires the prediction of a low-lying LUMO of dipolarophile [48]. Of significant interest is the possibility of predicting the difference in the frontier molecular orbitals (FMOs), from which the computational approach may be utilized. The FMO interaction of a HOMO_dipole_ pairing with LUMO_dipolarophile_ (or vice versa) was promoted, whereby a smaller energy gap emerged. With the goal of predicting such tentative evidence, four RGSs were calculated with incipient structural optimization (see Supporting Information Table S1) [37]. The azido model compound was computed in a previous report [49]. Figure 5 shows the predicted HOMO and LUMO energies of RGS.

The HOMO energy level difference between propargyl and 3-butynyl species (i.e., |*E*_HOMO, *n*=1_ − *E*_HOMO, *n*=2_|) was found to be 0.018 eV and 0.008 eV for the SH-based RGS and DSD-based RGS, respectively. In contrast to this minor HOMO energy level difference, a significant difference in LUMO energy level (i.e., |*E*_LUMO, *n*=1_ − *E*_LUMO, *n*=2_|) of approximately 0.070 eV was observed for both types of RGSs. Owing to the analogous HOMO energy level of propargyl and 3-butynyl species, the magnitude of the LUMO was actually responsible for determining the click reactivity [48]. The observed low-lying LUMO for propargyl species was indicative of faster reactivity, which has been verified experimentally. Figure 5 presents the calculated absolute values of the energy gap of the HOMO_RGS_ − LUMO_GAP_ (Δ*E*_1_) and HOMO_RGS_ − LUMO_GAP_ (Δ*E*_2_). The direct comparison of the FMO energy gap between RGS (*n* = 1) and RGS (*n* = 2), including the two-pathway interaction, suggested that the total energy gap difference, denoted as Σ*E*_3_, had been considered. The Σ*E*_3_ between RGS (*n* = 1) and RGS (*n* = 2) was obtained based on Equation (1), where the Δ*E*_1, *n*=1_, Δ*E*_1, *n*=2_, Δ*E*_2, *n*=1_ and Δ*E*_2, *n*=2_ were the Δ*E*_1_ and Δ*E*_2_ for propargyl and 3-butynyl species, respectively.

Σ*E*_3_ = (Δ*E*_1, *n*=2_ − Δ*E*_1, *n*=1_) + (Δ*E*_2, *n*=2_ − Δ*E*_2, *n*=1_) (1)

The Σ*E*_3_ values for the SH- and DSD-based RGS were calculated to be positive values of 0.088 and 0.078 eV, confirming that propargyl species had a smaller energy gap. On the other hand, the predicted Σ*E*_3_ was associated with an *E*_a_ difference between propargyl and 3-butynyl species, denoted as Δ*E*_4_. The values of Δ*E*_4_ were 0.082 eV for SH-based RGS and 0.075 eV for DSD-based RGS, with the conversion of the unit from kJ mol^−1^ to eV. Therefore, the theoretical prediction underlies the strong correlation with the experimental determination and offers an explicit explanation for the spacer-controlled Huisgen click reactivity.

### 2.5. Thermal Stability

Studies on the dual curing systems composed of a click reaction and a PU reaction showed that the two reactions have good chemical compatibility [49]. The RGS@GAP-based PU binders were fabricated and comprised molar ratios of [C≡C]/[N_3_] at 0.1/1 and 0.3/1. Figure S16 shows the TGA and DTG thermogram curves, where the temperature at the maximum thermal degradation rate (*T*_d, max_) was obtained (see Supporting Information). The observant *T*_d, max_, ranging from 188 to 229 °C with respect to the pure RGS, showed that they were less thermally stable than uncured GAP, which had a *T*_d, max_ of 248 °C [11]. As expected, early weight loss of the RGS@GAP-based PUs ranging from 188 to 229 °C was not observed, indicating that all RGSs had been thoroughly integrated into the polymeric matrix through the click reaction. On the other hand, it was also verified that much of the weight loss of the RGS was attributed to thermal evaporation rather than thermal decomposition due to the relatively low molecular weight. The *T*_d, max_ of all PU binders ranged from 222 to 241 °C, suggesting that sharp weight loss was ascribed to the thermal decomposition of the –N_3_ group [49,50,51,52].

### 2.6. Tensile Properties

**Figure 6:**
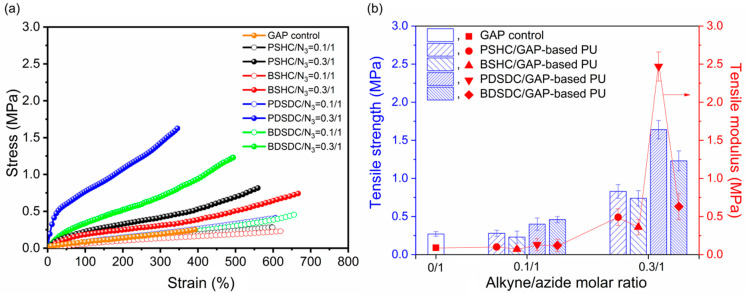
(**a**) Stress–strain curves and (**b**) comparison of the tensile properties of the RGS@GAP-based PUs.

Figure 6a presents the typical stress–strain curves and Figure 6b compares the summarized mechanical characteristics. For the composition at a 0.1/1 [C≡C]/[N_3_] stoichiometric ratio, the RGS@GAP-based PUs provided enhanced ultimate stress from 0.27 to 0.46 MPa and increased the ultimate elongation from 395% to 598–661% in comparison with the pristine PU. The Young’s modulus of RGS@GAP-based PUs ranged from 0.07 to 0.13 MPa, maintaining a similar level to the pristine GAP-based PU of 0.09 MPa. In cases where there is a low RGS content, the RGS@GAP-based PUs maintained similar tensile characteristics to the pristine GAP-based PU, suggesting that a low amount of RGS was unable to alter the mechanical characteristics of the pristine binder. At a 0.3/1 [C≡C]/[N_3_] stoichiometric ratio, the ultimate stress and Young’s modulus were increased to 0.74–1.64 MPa and 0.36–2.47 MPa, respectively. As the GAP-based PUs with a higher level of RGS incorporation exhibited more ductile manner, it is believed that the grafting extent of RGS determines the magnitude of the intermolecular interaction within the polymer chains. In particular, the PDSDC/GAP-based PU was initially subject to elastic deformation up to ca. 0.50 MPa, followed by plastic deformation with an elongation at break at 350%. In contrast to the PDSDC system, the BDSDC@GAP-based PU exhibited elastic behavior without a yield point. The miscibility study revealed the better molecular chain flexibility of the BDSDC@GAP binary mixture, resulting in a softer and more flexible nature for the BDSDC-incorporated PUs than the PDSDC-incorporated ones. The appreciable enhancement in tensile characteristics can be explained by the higher amount of triazole groups formed through the annulation of alkyne by azide increasing the resistance to the movement of the polymer skeleton and imparting higher strength [39]. The structure of the cyclic building blocks was also found to be another unneglectable factor in the enhancement of the mechanical characteristics. The RGS mounted on the pendant azide groups of the polymer backbone may withstand the motion of polymer chains, leading to concomitant improvement of the tensile characteristics, especially in terms of the bulky multi-perpendicular structure of DSD. The aforementioned study on the compatibility of the RGS/uncured GAP mixture showed that the flexibility dependent on the size of the building blocks can be ranked as SH > DSD. The tensile characteristics of the RGS@GAP-based PUs followed this trend. On the other hand, the GAP PUs plasticized by BTTN and TMETN presented decreased tensile properties when increasing the addition of plasticizers, which were affected by the processing effect [6,53].

### 2.7. Thermochemical Properties

**Figure 7:**
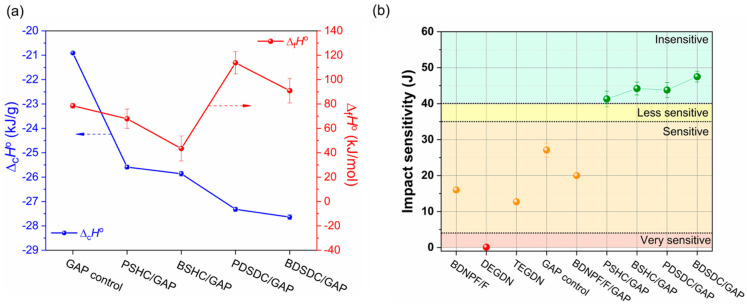
(**a**) Thermochemical characteristics and (**b**) impact sensitivity of RGS@GAP−based PUs.

The heat of combustion (Δ*_c_ H^o^*) of RGS@GAP-based PUs with a 0.3/1 [C≡C]/[N_3_] stoichiometric ratio was measured. As shown in Figure 7a, the measured Δ*_c_ H^o^* of the pristine PU was approximately −20.9 kJ g^−1^, which was in good agreement with the reported result [54]. The Δ*_c_ H^o^s* of the RGS@GAP-based PUs were higher than that of the pristine PU and increased with increasing carbon and hydrogen content (see Table S2 in Supporting Information) [55]. The Δ*_c_ H^o^* of SH-based GAP binders is ca. −25.7 kJ g^−1^ and that of DSD-based GAP binders is ca. −27.5 kJ g^−1^. The value of Δ*_c_ H^o^* depends primarily on the molar percentage of carbon and hydrogen in the samples because they consume O_2_ to completely convert it to CO_2_ and H_2_O during combustion along with releasing Δ*_c_ H^o^*. A lower molar percentage of carbon and hydrogen in the PSHC and PDSDC-based GAP binders leads to a lower Δ*_c_ H^o^* compared with their congeners that have a higher molecular weight. In addition, the increase in Δ*_c_ H^o^* for the RGS@GAP-based PUs was also composed of the elevated ring strain energies, which should be considered.

Δ*_f_ H^o^*, regarded as a critical factor for an energetic material, determines whether the reaction heat is negative or positive [55]. A negative Δ*_f_ H^o^* for the molecule (C_a_H_b_O_c_N_d_ in this study) shows that certain energy is liberated during the formation from its constituent elements. A positive Δ*_f_ H^o^* means the opposite situation, where certain energy for triggering the reaction is required. In other words, a positive Δ*_f_ H^o^* is favorable because of the higher energy output during the reaction. The Δ*_f_ H^o^* value of C_a_H_b_O_c_N_d_ can be calculated from the *Δ_c_ H^o^* results, and Equation (S5) is commonly used (see Supporting Information).

As reported, the GAP prepolymers with different molecular weights exhibited a similar experimental empirical formula [54]. The resulting GAP-based PUs showed large differences in their experimental empirical formulae because of the different amounts of curing agent. In this study, it was envisaged that all PU binders had the corresponding experimental empirical formula to be considered by the inclusion of RGS. The weight percentages of all elements of the RGS@GAP-based PUs at a 0.3/1 [C≡C]/[N_3_] stoichiometric ratio were determined by elemental analysis and are grouped in Table S2. The Δ*_f_ H^o^*s of all PUs were calculated using Equation (S5). As shown in Figure 7a, the reasonable Δ*_f_ H^o^* of 78.5 kJ mol^−1^ for the GAP control (~67.0 kJ mol^−1^ reported elsewhere [54]) was higher than that of 43.5–67.9 kJ mol^−1^ for the SH-based GAP binders but lower than 90.9–113.8 kJ mol^−1^ for the DSD-based GAP binders. Compared with the reported GAP control prepared from a lower *M*_n_ of GAP prepolymer, a higher Δ*_f_ H^o^* for the GAP control was obtained in this study because of the higher *M*_n_ of the GAP prepolymer, which required a lower content of curing agent (i.e., forming a lower content of hard segment). In particular, the building block in RGS is a reliable and promising energetic source that can be attributed to the RSE; the relief of which enhances the energetic performance significantly, particularly in terms of the DSD moiety.

### 2.8. Impact Sensitivity

The sensitivity of the PBX formulation is typically correlated with sensitive functional groups. A much-desired characteristic of a modern highly energetic material is that it is impact or shock insensitive, resulting in safer material handling. According to the classification standard of impact sensitivity for the transport of dangerous items [56], values greater than 40 J belong to an insensitive classification. TEGDN, BDNPF/F and GAP control exhibited impact sensitivities of 12.7, 16.0 and 27.1 J, respectively, which belong to the sensitive classification (4–35 J). The commonly known energetic plasticizer diethylene glycol dinitrate (DEGDN) has an impact sensitivity of 0.1 J, which means it is in the very sensitive category (<4 J) [57]. As shown in Figure 7b, the RGS@GAP-based PUs had an impact sensitivity of 41.3–47.5 J, which is a higher value than for the GAP control (27.1 J). The integration of RGS into GAP-based PUs is a rationalized approach to reducing the impact sensitivity via the formation of more stable triazole groups from the sensitive azide groups [11]. Further, the impact sensitivities of the RGS@GAP-based PUs were found to be higher than those for GAP-based PUs blended with BDNPF/F of 20 J [49] at the equivalent composition, indicating that those highly strained rings were less sensitive than the explosophore groups.

### 2.9. Migration Resistance

The migration resistance of RGS@GAP-based PUs with a 0.3/1 [C≡C]/[N_3_] stoichiometric ratio was determined to detect the presence of the unreacted RGS within the polymer matrix and the extractability of the reacted RGS. As reported, dimethylacetamide (DMA) was an ideal extraction medium for GAP-based PU to reach the maximum swelling [58]. The specimen was immersed in DMA and stirred for 2000 min to accelerate the RGS migration. According to the continuous swelling in DMA, the unreacted and detached items from the polymer matrix were detected by gas chromatography (GC). As shown in Figures S17 and S18, the signals for the pure RGS in DMA solution were undetectable in the GC spectra, verifying that all RGS was completely linked to the GAP matrix and achieved zero migration.

## 3. Experimental

### 3.1. Materials

All chemicals for the synthesis of RGS and GAP-based PUs are provided in the Supporting Information.

### 3.2. Characterization

The RGS and PU binders were successfully characterized using various techniques and the specific measurement conditions are included as Supporting Information.

### 3.3. Synthesis of RGS

Scheme 1 shows the synthetic route to RGS; all procedures are specified in the Supporting Information. The ^1^H and ^13^C NMR spectra of the resultant products for each step are presented in Figures S1–S4. The mass spectra of the RGSs are presented in Figures S5–S8.

### 3.4. Fabrication of RGS@GAP-Based PUs

**Scheme 2:**
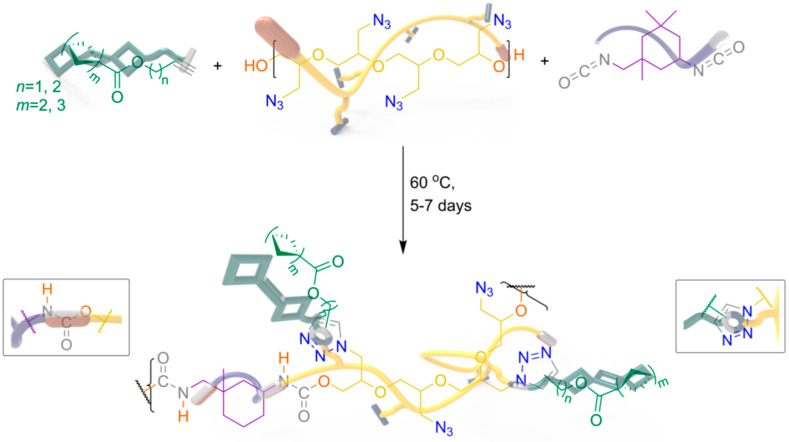
Fabrication of GAP-based PUs with grafting of RGS via in situ catalyst-free click chemistry.

Scheme 2 shows the reaction scheme to RGS@GAP-based PUs. The specific procedures for the fabrication of RGS@GAP-based PUs are provided in the Supporting Information.

## 4. Conclusions

Two types of highly ring-strained spirane hydrocarbons, SH and DSD, were embedded into RGS synthesized via multi-step procedure. The predicted RSEs of RGS using homodesmotic schemes were analogous to those of the corresponding numbers of 1,1-dimethylcyclobutane and were accompanied by a decrease of ca. 10 kJ mol^−1^ compared with their unsubstituted spiranes. RGS and GAP prepolymer were thermodynamically miscible within compositions up to 50/50 *w*/*w*. The quantitative miscibility indicated that the smaller building blocks with lower *I* values may impart better flexibility than uncured GAP. The isothermal viscosity measurements for the RGS/GAP mixtures revealed a significant decrease in the viscosity of uncured GAP, from 6000 to 100–170 cP at ambient temperature via the inclusion of an equal amount of RGS. No appreciable viscosity increases were observed in these mixtures after 300 min, which is indicative of the mild reactivity at the processing temperature. The click chemistry of RGS towards uncured GAP, which was initially studied by ^1^H NMR spectroscopy, showed that RGS (*n* = 1) exhibited higher reactivity. The RGS (*n* = 1) was determined to provide an *E*_a_ of 80.3–81.6 kJ mol^−1^, whereas RGS (*n* = 2) showed a higher *E*_a_ of 88.2–88.8 kJ mol^−1^. The rationalization of two experimental approaches showed that the Huisgen click reactivity with respect to *n* = 1 vs. *n* = 2 could be tunable because the degree of electron deficiency of the alkyne adjacent to the EWG was different. The computational results for the FMO energy gaps also showed that the propargyl-based dipolarophile with a low-lying LUMO_dipolarophile_ led to a faster reactivity. The total difference in the FMO energy gap was similar to the *E*_a_ difference, with a deviation of less than 1.0 kJ mol^−1^. RGS@GAP-based PUs displayed improved mechanical properties that were affected by the structures of the ring-strained building blocks and the amount of triazole groups stemmed from their sterically bulky moieties and high-affinity interactions within polymer chains. By grafting the ring-strained building blocks, the thermochemical characteristics of RGS@GAP-based PUs were significantly improved due to the higher energetic compensation originating from RSE. GAP-based PUs shifted to the insensitive region after grafting the RGS, which was ascribed to the newly formed triazoles.

## Figures and Tables

**Scheme 1:**
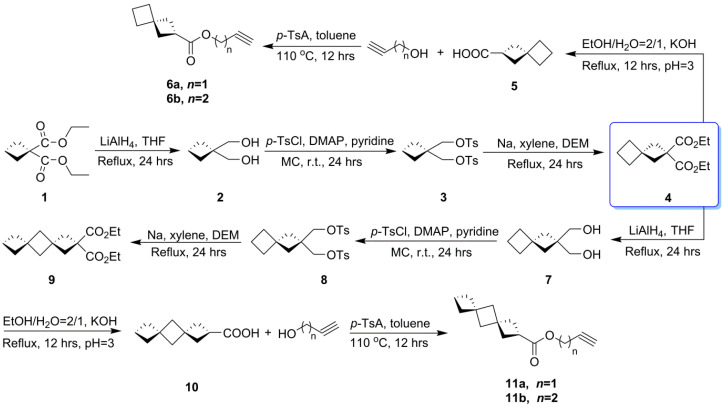
Synthetic route to SH- and DSD-based RGS.

## Data Availability

All data included in this study are available upon request by contact with the corresponding author.

## Supplementary Materials

The following supporting information can be downloaded at: https://www.mdpi.com/article/10.3390/polym15234564/s1, Figure S1: ^1^H NMR spectra of spiro[3.3]heptane-based RGSs; Figure S2: ^13^C NMR spectra of spiro[3.3]heptane-based RGSs; Figure S3: ^1^H NMR spectra of dispiro[3.1.3^6^.1^4^]decane-based RGSs; Figure S4: ^13^C NMR spectra of dispiro[3.1.3^6^.1^4^]decane-based RGSs; Figure S5: Mass spectrum of PSHC; Figure S6: Mass spectrum of BSHC; Figure S7: Mass spectrum of PDSDC; Figure S8: Mass spectrum of BDSDC; Figure S9: DSC thermograms of (a) GAP/PSHC, (b) GAP/BSHC, (c) GAP/PDSDC, and (d) GAP/BDSDC; Figure S10: Composition-dependent *T*_g_ of RGSs/GAP prepolymer binary mixtures; Figure S11: Viscosity *versus* time for binary mixtures of RGSs/GAP prepolymer (50/50 *w*/*w*) isothermally at 30 °C; Figure S12: Comparative ^1^H NMR spectra of the catalyst-free azide-alkyne 1,3-DPCA reaction with respect to (a) PSHC (*n* = 1) and (b) BSHC (*n* = 2) toward the GAP prepolymer performed under the bulk conditions at 60 °C, respectively; Figure S13: Comparative ^1^H NMR spectra of catalyst-free azide-alkyne 1,3-DPCA reaction with respect to (a) PDSDC (*n* = 1) and (b) BDSDC (*n* = 2) toward GAP prepolymer performed under the bulk condition at 60 °C, respectively; Figure S14: Degree of reaction *versus* reaction time plot for the catalyst-free azide-alkyne 1,3-DPCA reaction of the RGSs with the GAP prepolymer at 60 °C; Figure S15: Dynamic DSC curves of (a) PSHC (*n* = 1)/GAP and (b) BSHC (*n* = 2)/GAP mixtures at different heating rates; Figure S16: (a) TGA and (b) DTG curves of the RGSs and RGSs@GAP-based PUs; Figure S17: GC spectra for detection of unreacted RGSs in (a) PSHC@GAP-based PU and (b) BSHC@GAP-based PU; Figure S18: GC spectra for detection of unreacted RGSs in (a) PDSDC@GAP-based PU and (b) BDSDC@GAP-based PU; Table S1: LUMO and HOMO energy levels of RGS computed at the B3LYP/6-31G* level of theory; Table S2: Percentage of carbon (C), hydrogen (H), oxygen (O) and nitrogen (N), and empirical formula for the GAP control and RS/GAP-based PUs.
