# Supplementary material for: Simultaneous Enhancement of the Mechanical Properties, Performance and Insensitivity of an Energetic Elastomeric Polyurethane Binder by Kinetically Grafting Reactive Spiranes
Source: Polymers (Basel). 2023 Nov 28;15(23):4564. doi: 10.3390/polym15234564 (PMC10708326; doi:10.3390/polym15234564)
Supplement: Supplementary file 1 [file polymers-15-04564-s001.zip › polymers-2710341-supplementary.pdf]

Supporting Information

Simultaneous Enhancement of the Mechanical Properties, Performance and Insensitivity of an Energetic Elastomeric Polyurethane Binder by Kinetically Grafting Reactive Spiranes

Mingyang Ma [a] and Younghwan Kwon [b*]

[a] *School of Chemistry and Materials Science, East China University of Technology, Nanchang 330013, China*
[b]*Department of Chemical Engineering, Daegu University, Gyeongsan, Gyeongbuk 712-714, Korea*

*Corresponding author, email: y_kwon@daegu.ac.kr

Materials

Propargyl alcohol (99%), 3-butyn-1-ol (97%), *p*-toluenesulfonic acid monohydrate (*p*-TsA, 99%), lithium aluminum hydride ($LiAlH_4$, 95%), diethyl malonate (DEM, 99%), 4-(dimethylamino)pyridine (DMAP, 98%) and 3,5-dinitro salicylic acid (DNS, 98%) were purchased from Sigma-Aldrich Co. (USA). Diethyl cyclobutane-1,1-dicarboxylate (98%) and *p*-toluenesulfonyl chloride (*p*-TsCl, 98%) were supplied by Alfa Aesar Company. Toluene (99.8%), *p*-xylene (99.5%), tetrahydrofuran (THF, 99.5%), ethanol (EtOH, 95%), methylene chloride (MC, 99.5%), dimethylacetamide (DMA, 99.5%), sodium chloride (NaCl, 99.5%), pyridine (99.5%), sodium metal (99.8%), potassium hydroxide (KOH, 90%), magnesium sulfate ($MgSO_4$, 99.5%) and sodium bicarbonate ($NaHCO_3$, 99%) were supplied by Samchun Pure Chemical Co. Ltd. (Korea). Chloroform-d_1 ($CDCl_3$) (0.03 vol.%, TMS) and dimethyl sulfoxide-d_6 (DMSO-d_6) (0.03 vol.% TMS) were received from Merck Corp. GAP prepolymer (a hydroxyl equivalent weight of 2,000, 2.5–3 hydroxyl groups per molecule) was obtained from 3M Co.. Triphenyl bismuth (TPB, 98%) and isophorone diisocyanate (IPDI, 98%) were kindly donated from Agency for Defense Development.

Characterization

NMR spectra were recorded on a Varian-Mercury spectrometer (300 MHz). The chemical shifts were measured relative to $CDCl_3$ (H: δ = 7.26 ppm) or DMSO-d_6 (H: δ = 2.50 ppm) as the internal reference. The viscosity was obtained using a MCR 301 rheometer (Anton Paar Physica Co.). All experiments were held at 30 °C for 300 min at a constant shear rate of 1.0 s^{-1}. Glass transition temperature (T_g) was recorded by differential scanning calorimeter (DSC) (DSC8000, Perkin Elmer). All measurements were performed under nitrogen atmosphere at a heating rate of 10 °C min^{-1} ranging from –120 – 0 °C. Thermal stability was measured by thermogravimetric analyzer (TGA, SDTA851^e, Mettler Toledo) at a heating rate of 20 °C min^{-1} from 50 °C to 400 °C in nitrogen. The mechanical characteristics were measured on a texture analyzer (TA-HD+1500, Stable Micro Systems Ltd.) with a probe moving velocity of 0.8 mm s^{-1}. The tensile test specimens were made according to the specification (DIN 53504-Type S2). All tensile strengths, elongation at break and tensile moduli were the

averages of 3 measured values. The percentage of carbon (C), hydrogen (H) and nitrogen (N) of PU binders was obtained from a Perkin-Elmer 2400CHN analyzer. The percentage of oxygen (O) was calculated using following equation:

$$\%O = 100\% - \%C - \%H - \%N$$

Heat of combustion ($\Delta_c H^o$) was measured in an oxygen bomb calorimeter (Parr, model 1108P). Four tests were conducted for each sample. The impact sensitivity was obtained with an impact tester (Julius Peters) with a 10 kg weight drop hammer at 25 °C. According to the up–and–down method, the sensitivity is depicted as the impact sensitivity energy (J).

Synthesis of cyclobutane-1,1-diyldimethanol (2)

A mixture of $LiAlH_4$ (7.922 g, 0.209 mol) and anhydrous THF (150 mL) was mixed in a 250 mL two-necked round bottom flask equipped with a nitrogen inlet and condenser by magnetic stirrer at 0 °C. A solution of diethyl cyclobutane-1,1-dicarboxylate (Scheme 1) (20.000 g, 0.095 mol) and anhydrous THF (30 mL) was slowly dropwise added into the flask. After addition, the solution was refluxed for 24 hrs. The resulting solution was then slowly dropwise added with water until the excess of $LiAlH_4$ was reacted. The solution was washed with ethyl acetate 5 times. The organic extracts were dried over $MgSO_4$ and the solvent was removed by rotary evaporator to give the colorless viscous liquid (yield: 97.2%). ^{1}H NMR (300 MHz, $CDCl_3$): δ = 1.70 ppm (m, 4H, CH_2–C), 1.85 ppm (t, 2H, $C\underline{H_2}$–CH_2), 3.64 ppm (t, *J*=2.6 Hz, 4H, HO–$C\underline{H_2}$), 4.03 ppm (t, *J*=17.3 Hz, 2H, $\underline{H}$O–CH_2). ^{13}C NMR (75MHz, $CDCl_3$, ppm) δ = 15.56, 25.98, 43.32, 68.97. Elemental analysis ($C_6H_{12}O_2$, 116.16): Calcd, C, 62.04; H, 10.41; O, 27.55; Found, C, 62.01; H, 10.45; O, 27.54.

Synthesis of cyclobutane-1,1-diylbis(methylene) bis(4-methylbenzenesulfonate) (3)

A mixture of compound **2** (Scheme 1) (10.420 g, 0.090 mol), *p*-TsCl (37.623 g, 0.197 mol), pyridine (28.381 g, 0.359 mol) and DMAP (2.192 g, 0.018 mol) was dissolved in dichloromethane (180 mL). The solution was stirred in a 250 mL two-necked round bottom

flask equipped with a nitrogen inlet and condenser at room temperature for 24 hrs. After reaction, the solution was washed by saturated NaCl solution three times. The organic extracts were dried over $MgSO_4$ and the solvent was removed by rotary evaporator to give the white powder (yield: 91.1%). ^{1}H NMR (300 MHz, $CDCl_3$): δ = 1.92–1.75 ppm (m, 2H, 2H, 2H, $C\underline{H}_2$–CH_2, CH_2–C, CH_2–C), 2.45 ppm (s, *J*=1.4 Hz, 6H, –CH_3), 3.93 ppm (s, 4H, CH_2–O), 7.35 ppm (d, *J*=7.4 Hz, 2H, =$C\underline{H}$–C–CH_3), 7.70 ppm (d, *J*=8.4 Hz, 2H, $C\underline{H}$=CH–C). ^{13}C NMR (75MHz, $CDCl_3$, ppm) δ =15.15, 21.88, 25.49, 40.82, 71.89, 128.04, 130.12, 132.49, 145.33. Elemental analysis ($C_{20}H_{24}O_6S_2$, 424.53): Calcd, C, 56.59; H, 5.70; O, 22.61; S, 15.10; Found, C, 56.57; H, 5.73; O, 22.64; S, 15.06.

Synthesis of diethyl spiro[3.3]heptane-2,2-dicarboxylate (4)

A mixture of diethyl malonate (30.722 g, 0.192 mol), sodium metal (4.412 g, 0.192 mol) and *p*-xylene (180 mL) was heated to form the sodium salt in a 250 mL two-necked round bottom flask equipped with a nitrogen inlet and condenser. Compound **3** (Scheme 1) (32.69 g, 0.077 mol) was then added and the solution was refluxed for 24 hrs. After reaction, the solution was washed by sat. aq $NaHCO_3$ solution three times. The organic extracts were dried over $MgSO_4$ and the solvent was removed by rotary evaporator. The final crude product was purified by column chromatography (silica gel, ethyl acetate/hexane (v/v) = 1/10) to give light yellow liquid (yield: 83.4%). ^{1}H NMR (300 MHz, $CDCl_3$): δ = 1.16 ppm (m, 6H, CH_3), 1.69 ppm (m, 2H, CH_2–$C\underline{H}_2$), 1.91 ppm (t, 4H, CH_2–C–), 2.47 ppm (t, 4H, CH_2–C–), 4.11 ppm (m, *J*=8.0 Hz, 4H, O–CH_2). ^{13}C NMR (75MHz, $CDCl_3$, ppm): δ =14.28, 16.27, 35.29, 38.59, 41.63, 48.82, 61.55, 172.17. Elemental analysis ($C_{13}H_{20}O_4$, 240.30): Calcd, C, 64.98; H, 8.39; O, 26.63; Found, C, 64.95; H, 8.37; O, 26.68.

Synthesis of spiro[3.3]heptane-2-carboxylic acid (5)

A mixture of compound **4** (Scheme 1) (10.000 g, 0.042 mol), EtOH (100 mL), H_2O (50 mL) and KOH (9.340 g, 0.166 mol) was charged to a 250 mL one-necked round bottom flask and refluxed for 12 hrs. Then, the solution was cooled down to room temperature and adjusted pH to 3. The solution was transferred into a separatory funnel with the aid of ethyl acetate. The

ethyl acetate solution was washed with water 3 times. The raw product was obtained by removing the solvent and subsequently dissolved in pyridine refluxing for 24 hrs. The solution was dried over $MgSO_4$ and the product was obtained by removing solvents to yield 83.2%. ^{1}H NMR (300 MHz, DMSO-d_6): δ = 1.71–2.09 ppm (m, 2H, 2H, 2H, 2H, 2H, $C\underline{H}_2$–CH_2, CH_2–C, CH_2–C, $C\underline{H}_2$–CH, $C\underline{H}_2$–CH), 2.84 ppm (m, *J*=7.0 Hz, 1H, C$\underline{H}$–COOH). ^{13}C NMR (75 MHz, DMSO-d_6, ppm): δ =16.48, 32.92, 34.78, 35.28, 38.02, 40.20, 176.91. Elemental analysis ($C_8H_{12}O_2$, 140.18): Calcd, C, 68.54; H, 8.63; O, 22.83; Found, C, 68.52; H, 8.64; O, 22.84.

Synthesis of prop-2-yn-1-yl spiro[3.3]heptane-2-carboxylate (6a) and but-3-yn-1-yl spiro[3.3]heptane-2-carboxylate (6b)

A mixture of compound **5** (Scheme 1) (2.536 g, 0.018 mol), propargyl alcohol or 3-butyn-1-ol (1.512 g or 1.890 g, 0.027 mol), *p*-TsA (0.384 g, 0.002 mol), and toluene (70 mL) was heated to 110 °C in a 100 mL one-necked round bottom flask equipped with the Dean-Stark trap and condenser refluxing for 12 hrs. After reaction, the toluene solution was cooled down, and washed with 10 wt.% $NaCO_3$ solution and water. Then, the toluene solution was dried over $MgSO_4$. The toluene solution was filtered and toluene was removed by rotary evaporator to give the crude liquid product. The final crude product was further purified by column chromatography (silica gel, ethyl acetate/hexane (v/v) = 1/10) to give the light yellow liquid PSHC (6a) and BSHC (6b). PSHC (*n*=1): Yield 80.0%. ^{1}H NMR (300 MHz, $CDCl_3$): δ = 1.71–2.22 ppm (m, 2H, 2H, 2H, 2H, 2H, $C\underline{H}_2$–CH_2, CH_2–C–, CH_2–C, $C\underline{H}_2$–CH, $C\underline{H}_2$–CH), 2.45 ppm (t, *J*=2.9 Hz, 1H, ≡C–H), 2.99 ppm (m, *J*=7.0 Hz, 1H, CH–COO), 4.63 ppm (m, *J*=2.9 Hz, 2H, O–$C\underline{H}_2$). ^{13}C NMR (75 MHz, $CDCl_3$, ppm): δ =16.37, 32.86, 34.59, 35.28, 40.45, 52.02, 74.94, 78.06, 174.86. BSHC (*n*=2): Yield 76.4%. ^{1}H NMR (300 MHz, $CDCl_3$): δ = 1.71–2.22 ppm (m, 2H, 2H, 2H, 2H, 2H, $C\underline{H}_2$–CH_2, CH_2–C–, CH_2–C, $C\underline{H}_2$–CH, $C\underline{H}_2$–CH), 1.96 ppm (t, 1H, ≡C–H), 2.45 ppm (t, *J*=4.4 Hz, 2H, CH_2–C≡), 2.98 ppm (m, *J*=7.0 Hz, 1H, CH–COO), 4.10 ppm (m, *J*=4.4 Hz, 2H, O–CH_2). ^{13}C NMR (75 MHz, $CDCl_3$, ppm): δ =16.36, 19.15, 33.03, 34.62, 35.29, 38.02, 40.43, 62.04, 70.06, 80.23, 175.42.

Synthesis of spiro[3.3]heptane-2,2-diyldimethanol (7)

Compound **7** was obtained by repeating the same synthetic route to compound **2** with a yield of 91.2% (Scheme 1). ^{1}H NMR (300 MHz, $CDCl_3$): δ = 1.80 ppm (m, *J*=10.6 Hz, 2H, $C\underline{H}_2$–CH_2), 1.96 ppm (t, *J*=7.1 Hz, 4H, CH_2–C), 2.05 ppm (t, 4H, CH_2–C), 3.62 ppm (t, *J*=1.0 Hz, 4H, HO–$C\underline{H}_2$). ^{13}C NMR (75MHz, $CDCl_3$, ppm) δ = 16.7, 36.8, 38.1, 38.6, 39.7, 70.3. Elemental analysis ($C_9H_{16}O_2$, 156.22): Calcd, C, 69.19; H, 10.33; O, 20.48; Found, C, 69.20; H, 10.34; O, 20.46.

Synthesis of spiro[3.3]heptane-2,2-diylbis(methylene) bis(4-methylbenzenesulfonate) (8)

Compound **8** was obtained by repeating the same synthetic route to compound **3** with a yield of 83.1% (Scheme 1). ^{1}H NMR (300 MHz, $CDCl_3$): δ = 1.90–1.75 ppm (m, 2H, 2H, 2H, $C\underline{H}_2$–CH_2, CH_2–C, CH_2–C), 2.01 ppm (t, 4H, CH_2–C–), 2.45 ppm (s, *J*=1.4 Hz, 6H, –CH_3), 3.92 ppm (s, 4H, CH_2–O), 7.35 ppm (d, *J*=8.2 Hz, 2H, =$C\underline{H}$–C–CH_3), 7.70 ppm (d, *J*=8.4 Hz, 2H, $C\underline{H}$=CH–C–). ^{13}C NMR (75MHz, $CDCl_3$, ppm) δ = 14.4, 15.2, 21.9, 25.6, 40.9, 60.6, 71.9, 128.1, 130.2, 132.6, 145.3. Elemental analysis ($C_{23}H_{28}O_6S_2$, 464.59): Calcd, C, 59.46; H, 6.08; O, 20.66; S, 13.80; Found, C, 59.45; H, 6.09; O, 20.64; S, 13.82.

Synthesis of diethyl dispiro[3.1.3^6.1^4]decane-2,2-dicarboxylate (9)

Compound **9** was obtained by repeating the same synthetic route to compound **4** with a yield of 73.4% (Scheme 1). ^{1}H NMR (300 MHz, $CDCl_3$): δ = 1.18 ppm (m, *J*=7.1 Hz, 6H, CH_3), 1.71 ppm (m, *J*=6.9 Hz, 2H, $C\underline{H}_2$–CH_2), 1.84 ppm (t, 4H, CH_2–C), 1.95 ppm (t, 4H, CH_2–C–), 2.46 ppm (t, 4H, CH_2–C), 4.12 ppm (m, *J*=7.2 Hz, 4H, O–CH_2). ^{13}C NMR (75MHz, $CDCl_3$, ppm): δ = 14.2, 16.8, 33.9, 35.3, 38.9, 41.8, 47.8, 49.2, 61.4, 172.0. Elemental analysis ($C_{16}H_{24}O_4$, 280.36): Calcd, C, 68.54; H, 8.63; O, 22.83; Found, C, 68.56; H, 8.62; O, 22.82.

Synthesis of dispiro[3.1.3^6.1^4]decane-2-carboxylic acid (10)

Compound **10** was obtained by repeating the same synthetic route to compound **5** with a yield of 71.4% (Scheme 1). ^{1}H NMR (300 MHz, $CDCl_3$): δ = 1.75–2.16 ppm (m, 2H, 2H, 2H, 2H, 2H, 2H, 2H, $C\underline{H}_2$–CH_2, CH_2–C, CH_2–C, CH_2–C, CH_2–C, $C\underline{H}_2$–CH, $C\underline{H}_2$–CH), 3.00 ppm (m,

J=7.0 Hz, 1H, C<u>H</u>–COOH). ^{13}C NMR (75 MHz, $CDCl_3$, ppm): δ = 16.9, 29.9, 34.6, 38.2, 47.8, 52.2, 181.8. Elemental analysis ($C_{11}H_{16}O_2$, 180.25): Calcd, C, 73.30; H, 8.95; O, 17.75; Found, C, 73.32; H, 8.91; O, 17.77.

Synthesis of prop-2-yn-1-yl dispiro[3.1.3^6.1^4]decane-2-carboxylate (11a) and but-3-yn-1-yl dispiro[3.1.3^6.1^4]decane-2-carboxylate (11b)

Compound **11a** (PDSDC) and **11b** (BDSDC) were obtained by repeating the same synthetic route to compound **6a** and **6b** (Scheme 1). PDSDC (n=1): Yield 81.8%. ^{1}H NMR (300 MHz, $CDCl_3$): δ = 1.71–1.99 ppm (m, 2H, 2H, 2H, 2H, 2H, C<u>H</u>$_2$–CH_2, CH_2–C, CH_2–C, CH_2–C, CH_2–C), 2.21 ppm (m, 4H, C<u>H</u>$_2$–CH), 2.44 ppm (t, 1H, ≡C–H), 2.99 ppm (m, J=8.5 Hz, 1H, C<u>H</u>–COO), 4.60 ppm (m, J=8.0 Hz, 2H, O–CH_2). ^{13}C NMR (75 MHz, $CDCl_3$, ppm): δ = 16.9, 33.3, 35.0, 35.4, 38.2, 47.2, 47.7, 52.0, 74.9, 78.1, 174.8. BDSDC (n=2): Yield 83.4%. ^{1}H NMR (300 MHz, $CDCl_3$): δ = 1.71–1.99 ppm (m, 2H, 2H, 2H, 2H, 2H, C<u>H</u>$_2$–CH_2, CH_2–C, CH_2–C, CH_2–C, CH_2–C), 1.94 ppm (t, 1H, ≡C–H), 2.21 ppm (m, 4H, C<u>H</u>$_2$–CH), 2.38 ppm (m, J=7.6 Hz, 2H, CH_2–C≡), 2.98 ppm (m, J=8.5 Hz, 1H, C<u>H</u>–COO), 4.07 ppm (m, J=7.1 Hz, 2H, O–CH_2). ^{13}C NMR (75 MHz, $CDCl_3$, ppm): δ = 16.9, 19.1, 33.5, 35.0, 35.4, 38.3, 46.8, 47.0, 62.0, 70.1, 80.2, 175.3.

Preparation of the RGSs@GAP-based PUs

The pre-drying treatment on the GAP prepolymer was performed at 80 °C under vacuum for 12 hrs prior to use. After that, GAP prepolymer (10.000 g, 1.81 mmol) was stirred at 60 °C under vacuum for 1 hr and cooled down to 30 °C with the continuous mixing for 0.5 hr. The vacuum was released and IPDI (0.564 g, 2.54 mmol) was added. After stirring for 0.5 hr, the predetermined amount of RGSs dependent on the molar ratio of [C≡C]/[N_3], two catalysts TPB (0.03 g, 20 wt.% in benzene) and DNS (0.06 g, 12.5 wt.% in benzene) was added and stirred for another 0.5 hr under vacuum. The mixture was cast on a Teflon coated mold (5 cm × 8 cm), held in a vacuum oven at 30 °C for 3 hrs, and finally subject to curing. The curing process was conducted in an oven at 60 °C for 5-7 days.

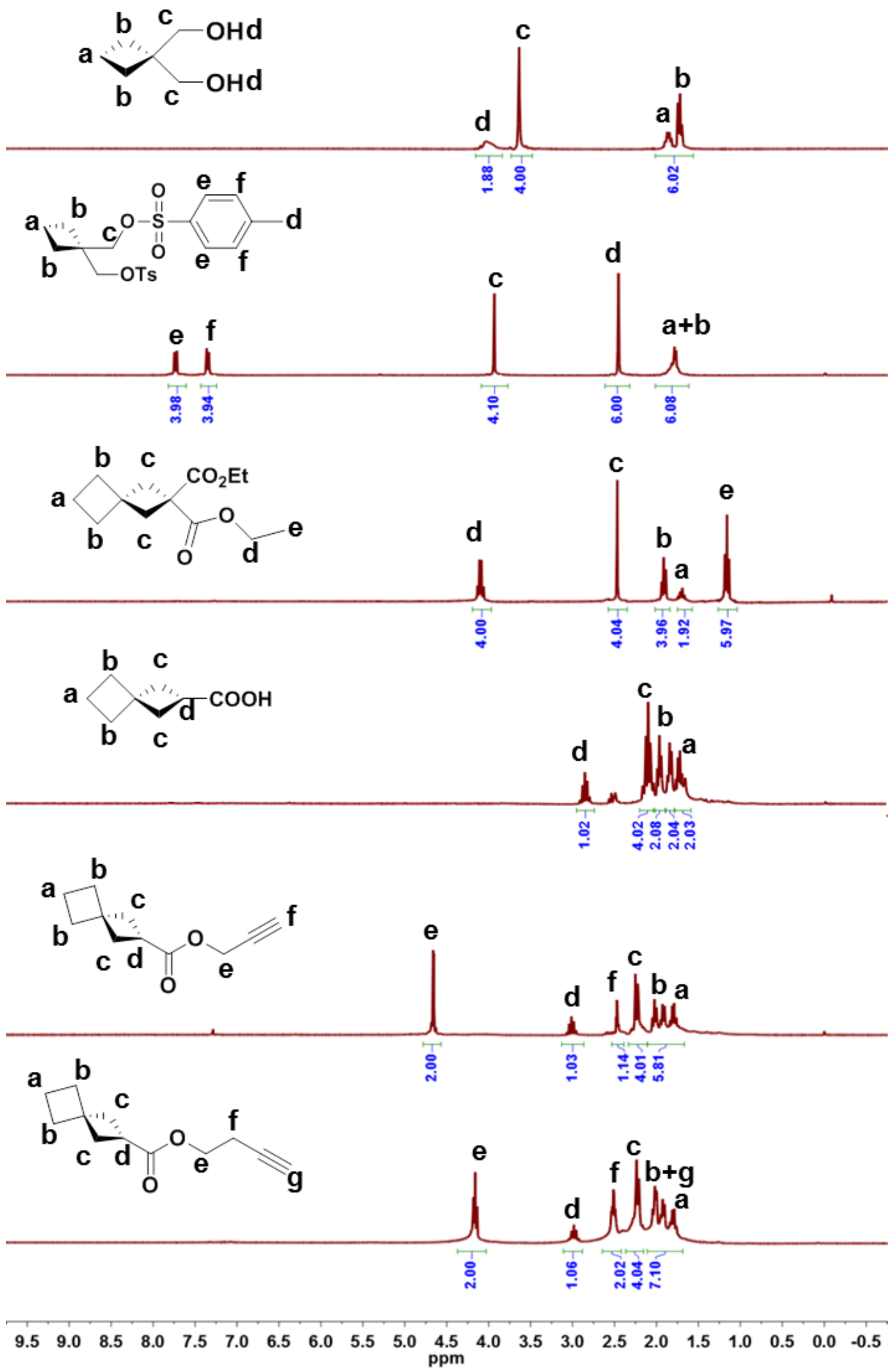

Fig. S1. ^{1}H NMR spectra of spiro[3.3]heptane-based RGSs.

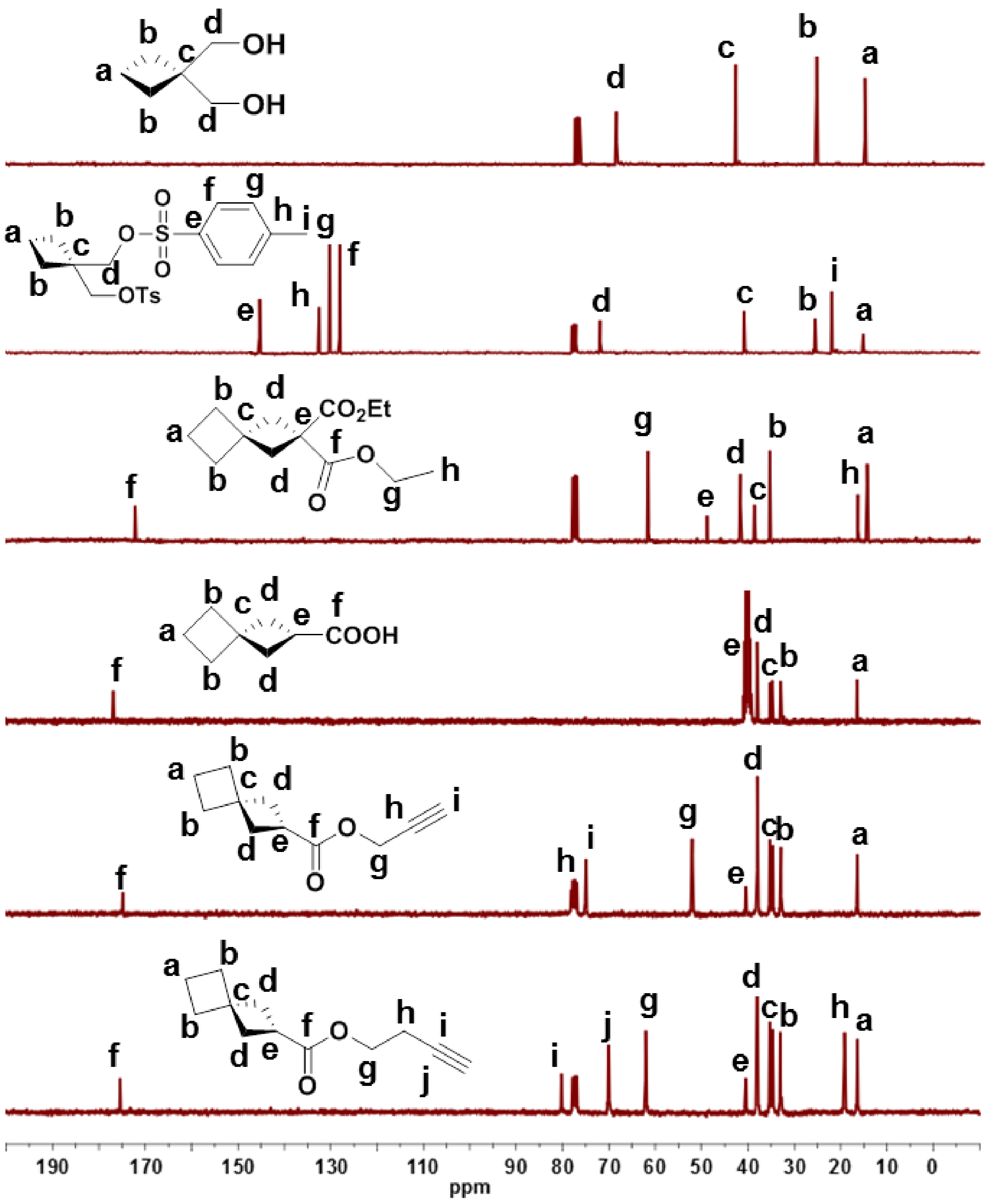

Fig. S2. ^{13}C NMR spectra of spiro[3.3]heptane-based RGSs.

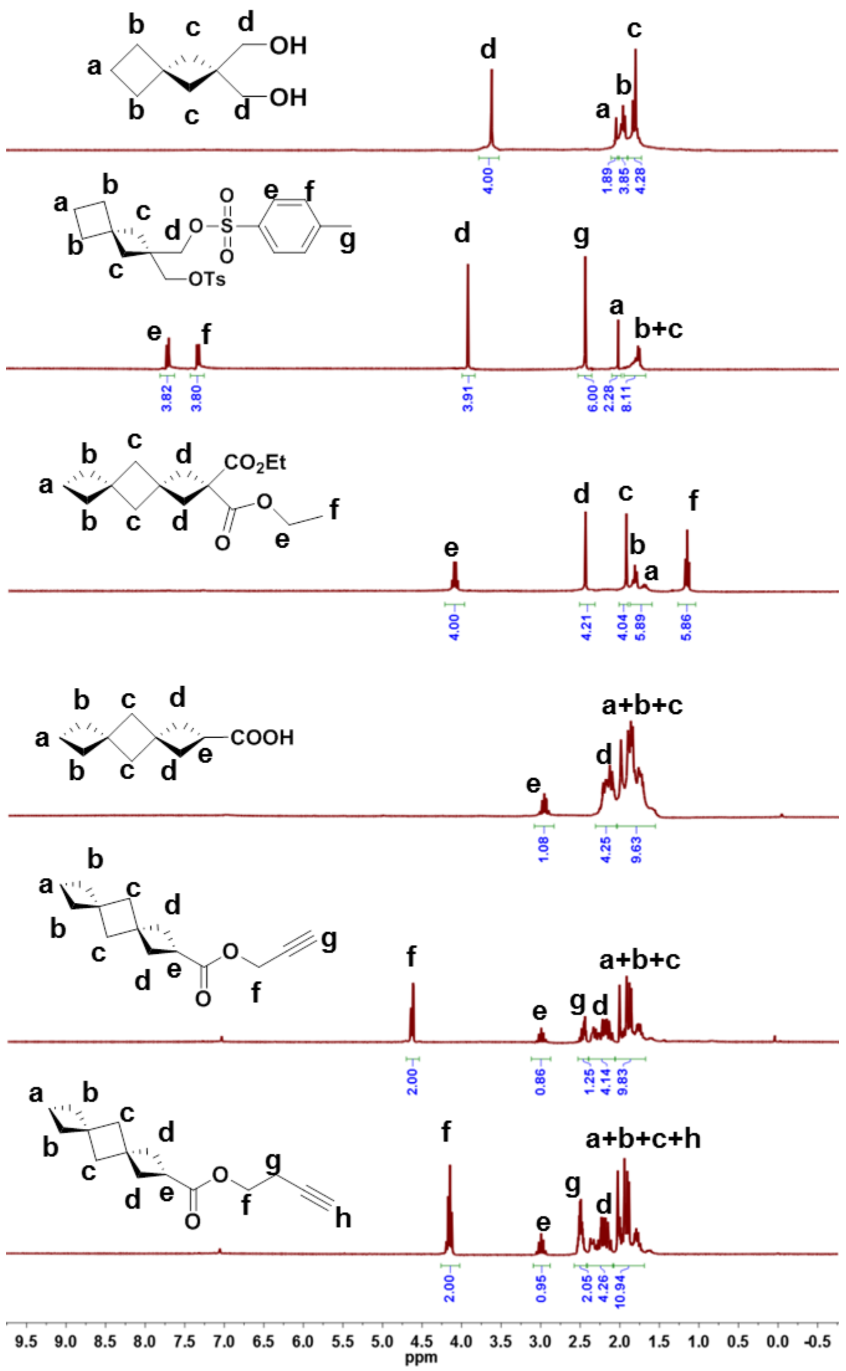

Fig. S3. ^{1}H NMR spectra of dispiro[3.1.3^6.1^4]decane-based RGSs.

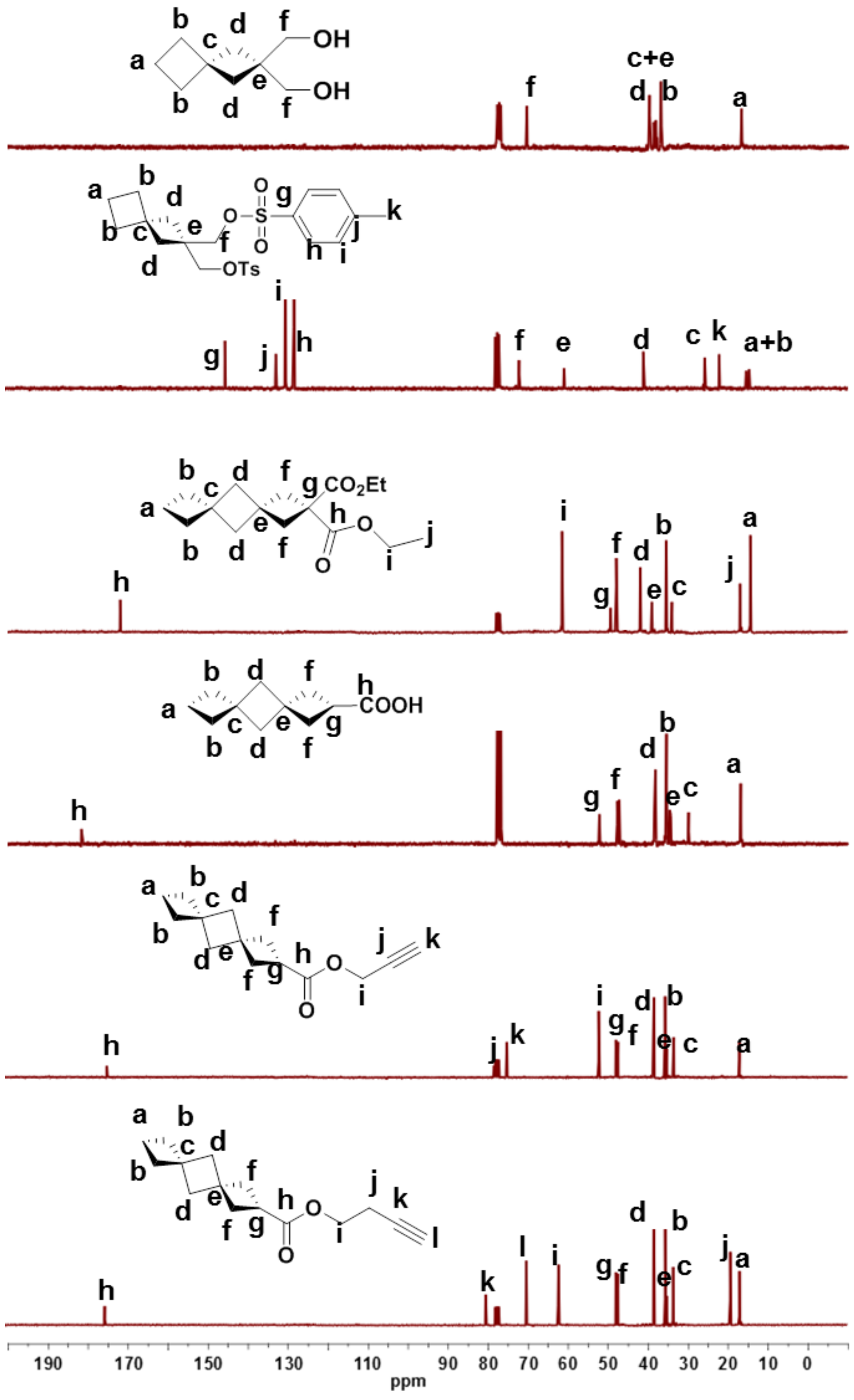

Fig. S4. ^{13}C NMR spectra of dispiro[3.1.3^{6}.1^{4}]decane-based RGSs.

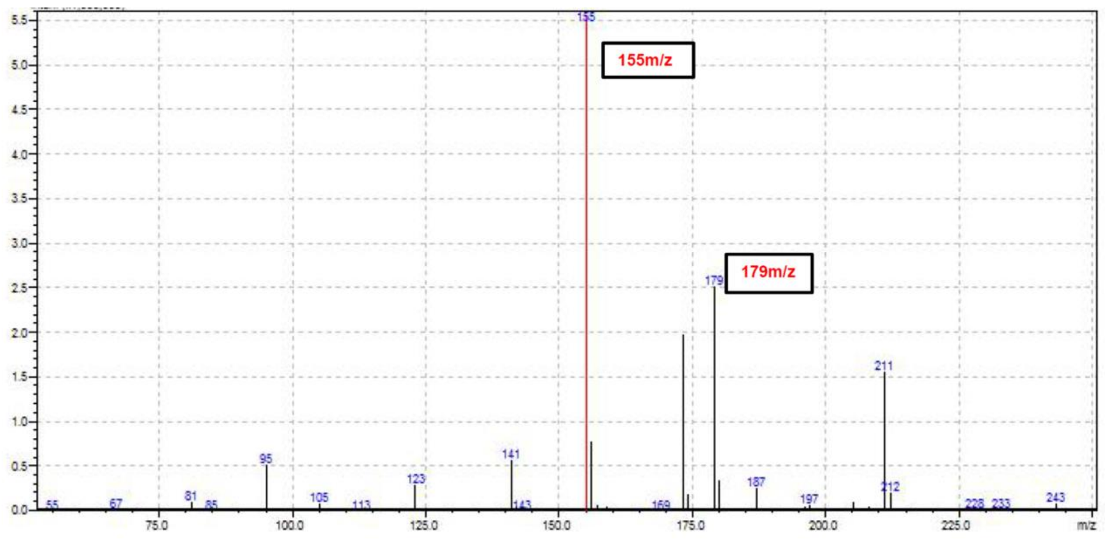

Fig. S5. Mass spectrum of PSHC.

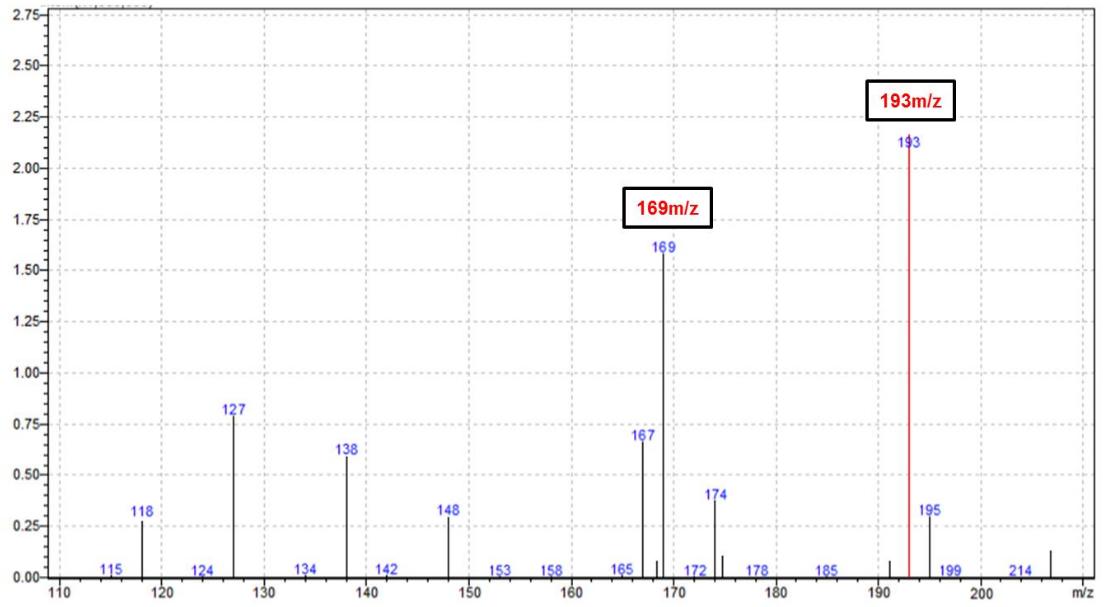

Fig. S6. Mass spectrum of BSHC.

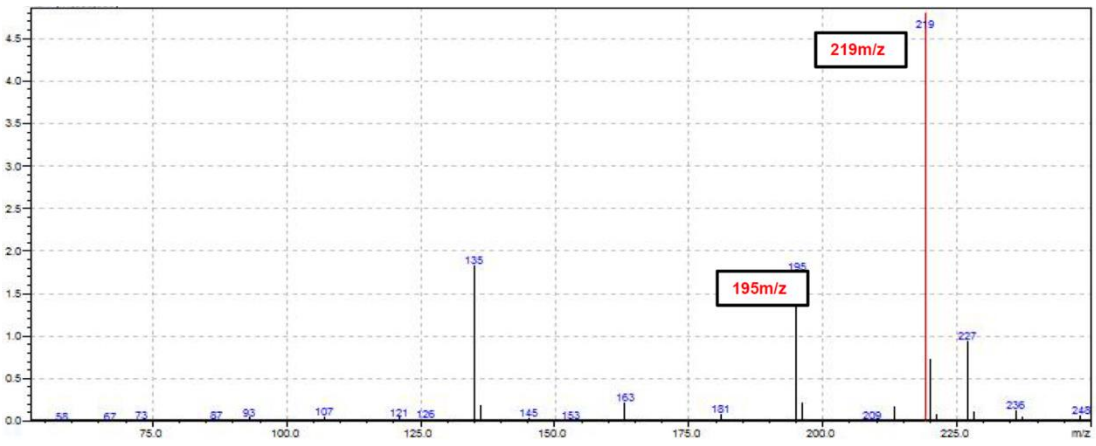

Fig. S7. Mass spectrum of PDSDC.

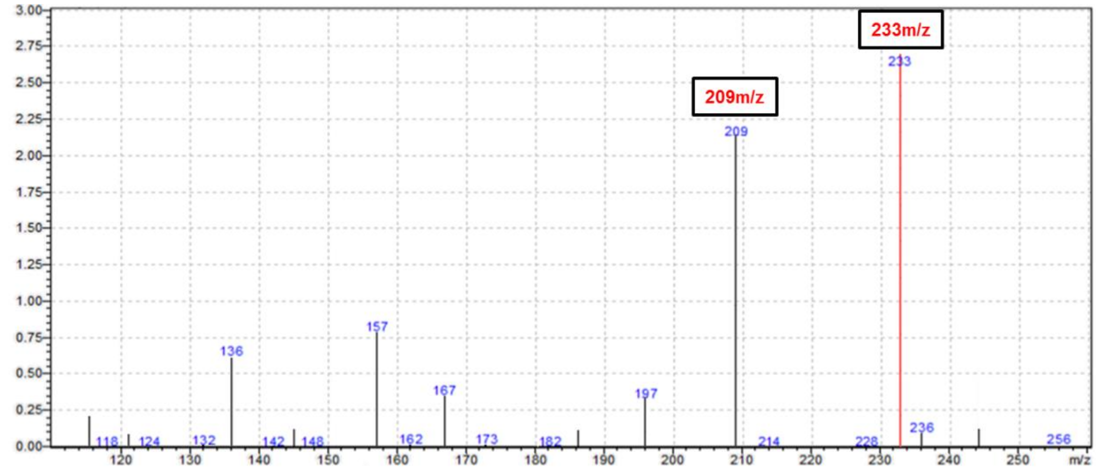

Fig. S8. Mass spectrum of BDSDC.

B3LYP/6-31G* Total Energies in hartrees.
Cartesian coordinates in Å.
All structures have zero imaginary frequencies.
The second column refers to the atom type: 1=hydrogen, 6=carbon, etc.

PSHC

E(RB+HF-LYP) = -577.9272789

Sum of electronic and zero-point Energies= -577.700694

1	6	0	-4.305555	1.825397	0.000000
2	6	0	-2.762369	3.368587	0.000000
3	6	0	-4.305598	3.368584	-0.000270
4	1	0	-4.840266	1.290561	-0.638694
5	1	0	-4.649227	1.481429	1.154145
6	1	0	-2.418434	3.712342	-1.154163
7	1	0	-2.227918	3.903450	0.638804
8	1	0	-4.758297	3.821113	-0.907235
9	1	0	-4.758868	3.821847	0.905959
10	6	0	-2.762308	1.825397	0.000000
11	6	0	-1.990567	1.054252	1.091495
12	6	0	-1.990718	1.053483	-1.090974
13	6	0	-1.218793	0.282537	0.000507
14	1	0	-2.631484	0.413179	1.732094
15	1	0	-1.349804	1.695673	1.731930
16	1	0	-2.631961	0.411927	-1.730909
17	1	0	-1.350030	1.694239	-1.732147
18	1	0	-1.406823	-0.811446	0.000936
19	6	0	0.298886	0.543788	0.000098
20	8	0	0.919795	1.605450	0.030203
21	8	0	1.019098	-0.605582	-0.040538
22	6	0	2.420624	-0.321635	-0.039569
23	1	0	2.674958	0.216193	0.849787
24	1	0	2.665183	0.270403	-0.896647
25	6	0	3.211923	-1.642007	-0.084984
26	6	0	3.825951	-2.666581	-0.120224
27	1	0	4.371126	-3.576266	-0.151513

BSHC

E(RB+HF-LYP) = -617.2471018

Sum of electronic and zero-point Energies= -616.991696

1	6	0	-2.556657	-0.906516	0.000000
2	6	0	-1.013471	0.636674	0.000000
3	6	0	-2.556700	0.636671	-0.000270
4	1	0	-3.091368	-1.441351	-0.638694
5	1	0	-2.900329	-1.250483	1.154145
6	1	0	-0.669536	0.980430	-1.154163
7	1	0	-0.479020	1.171537	0.638804
8	1	0	-3.009399	1.089200	-0.907235
9	1	0	-3.009970	1.089934	0.905959
10	6	0	-1.013410	-0.906516	0.000000
11	6	0	-0.241669	-1.677661	1.091495
12	6	0	-0.241819	-1.678429	-1.090974
13	6	0	0.530105	-2.449376	0.000507
14	1	0	-0.882586	-2.318733	1.732094
15	1	0	0.399094	-1.036239	1.731930
16	1	0	-0.883062	-2.319986	-1.730909
17	1	0	0.398869	-1.037674	-1.732147
18	1	0	0.342075	-3.543358	0.000936
19	6	0	2.047784	-2.188124	0.000098
20	8	0	2.668693	-1.126462	0.030203
21	8	0	2.767997	-3.337495	-0.040538
22	6	0	4.169522	-3.053547	-0.039569
23	1	0	4.423857	-2.515719	0.849787
24	1	0	4.414081	-2.461509	-0.896647
25	6	0	4.960821	-4.373919	-0.084984
26	1	0	4.707407	-4.911174	-0.974949
27	1	0	4.715335	-4.966491	0.771460
28	6	0	6.470165	-4.068185	-0.082151
29	6	0	7.641377	-3.830944	-0.079953
30	1	0	8.681256	-3.620305	-0.078001

PDSDC

E(RB+HF-LYP) = -694.6304696

Sum of electronic and zero-point Energies= -694.341030

1	6	0	-4.285714	1.306122	0.000000
2	6	0	-2.742528	2.849312	0.000000
3	6	0	-4.285757	2.849309	-0.000270
4	1	0	-4.820425	0.771287	-0.638694
5	1	0	-4.629386	0.962155	1.154145
6	1	0	-2.398593	3.193068	-1.154163
7	1	0	-2.208077	3.384175	0.638804
8	1	0	-4.738456	3.301838	-0.907235
9	1	0	-4.739027	3.302572	0.905959
10	6	0	-2.742467	1.306122	0.000000
11	6	0	-1.970726	0.534977	1.091495
12	6	0	-1.970877	0.534209	-1.090974
13	1	0	-2.422424	0.083287	1.847628
14	1	0	-1.154980	1.351451	1.577741
15	1	0	-2.787047	-0.282344	-1.576661
16	1	0	-1.519464	0.985525	-1.847617
17	6	0	-1.198952	-0.236738	0.000507
18	6	0	0.344295	-0.235859	0.000619
19	6	0	-1.198116	-1.779925	0.000777
20	6	0	0.345113	-1.779049	0.000619
21	1	0	0.796879	0.217212	0.907266
22	1	0	0.796983	0.217454	-0.905879
23	1	0	-1.650623	-2.232711	0.907709
24	1	0	-1.651062	-2.233446	-0.905485
25	1	0	0.798231	-2.231545	0.907307
26	6	0	0.973683	-2.407374	-1.257041
27	8	0	0.777725	-2.163160	-2.446798
28	8	0	1.876225	-3.374119	-0.953343
29	6	0	2.436424	-3.931948	-2.144940
30	1	0	2.926787	-3.161838	-2.702950
31	1	0	1.656569	-4.361145	-2.738671
32	6	0	3.456366	-5.023348	-1.770553
33	6	0	4.247814	-5.870245	-1.480039
34	1	0	4.950515	-6.622177	-1.222101

BDSDC

E(RB+HF-LYP) = -733.9502914

Sum of electronic and zero-point Energies= -733.9502914

1	6	0	-3.009868	-0.296053	0.000000
2	6	0	-1.466682	1.247137	0.000000
3	6	0	-3.009911	1.247134	-0.000270
4	1	0	-3.544579	-0.830888	-0.638694
5	1	0	-3.353541	-0.640020	1.154145
6	1	0	-1.122747	1.590893	-1.154163
7	1	0	-0.932231	1.782000	0.638804
8	1	0	-3.462610	1.699663	-0.907235
9	1	0	-3.463181	1.700397	0.905959
10	6	0	-1.466621	-0.296053	0.000000
11	6	0	-0.694880	-1.067198	1.091495
12	6	0	-0.695031	-1.067966	-1.090974
13	1	0	-1.146578	-1.518888	1.847628
14	1	0	0.120866	-0.250724	1.577741
15	1	0	-1.511201	-1.884519	-1.576661
16	1	0	-0.243618	-0.616650	-1.847617
17	6	0	0.076894	-1.838913	0.000507
18	6	0	1.620141	-1.838034	0.000619
19	6	0	0.077730	-3.382100	0.000777
20	6	0	1.620959	-3.381224	0.000619
21	1	0	2.072725	-1.384963	0.907266
22	1	0	2.072829	-1.384721	-0.905879
23	1	0	-0.374777	-3.834886	0.907709
24	1	0	-0.375216	-3.835621	-0.905485
25	1	0	2.074077	-3.833720	0.907307
26	6	0	2.249529	-4.009549	-1.257041
27	8	0	2.053571	-3.765335	-2.446798
28	8	0	3.152071	-4.976294	-0.953343
29	6	0	3.712270	-5.534123	-2.144940
30	1	0	4.202633	-4.764013	-2.702950
31	1	0	2.932415	-5.963320	-2.738671
32	6	0	4.732212	-6.625523	-1.770553
33	1	0	5.511231	-6.196650	-1.175492
34	1	0	4.241518	-7.396409	-1.213907
35	6	0	5.337180	-7.224678	-3.053763
36	6	0	5.806619	-7.689607	-4.049500
37	1	0	6.223418	-8.102401	-4.933582

The homodesmotic reactions were constructed from the equilibrium number of atoms and bond types in strain and strain-free terms, as shown in Eq. S1 and S2,

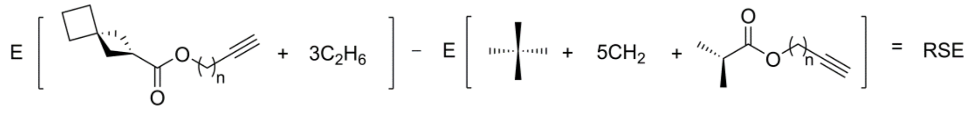

(S1)

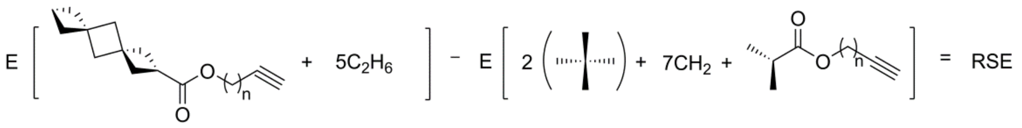

(S2)

where E is the computed total energy. C_2H_6 was used in the left term to offset the methyl groups due to the cleavage of spirane generating the congeneric acyclic molecule with a methyl fragment. The $E(CH_2)$ obtained from the total energy difference between (n+1) and (n) acyclic alkane, *e. g.*, E(n-butane) – E(propane), rationalized the number of methylene in the two energy terms.

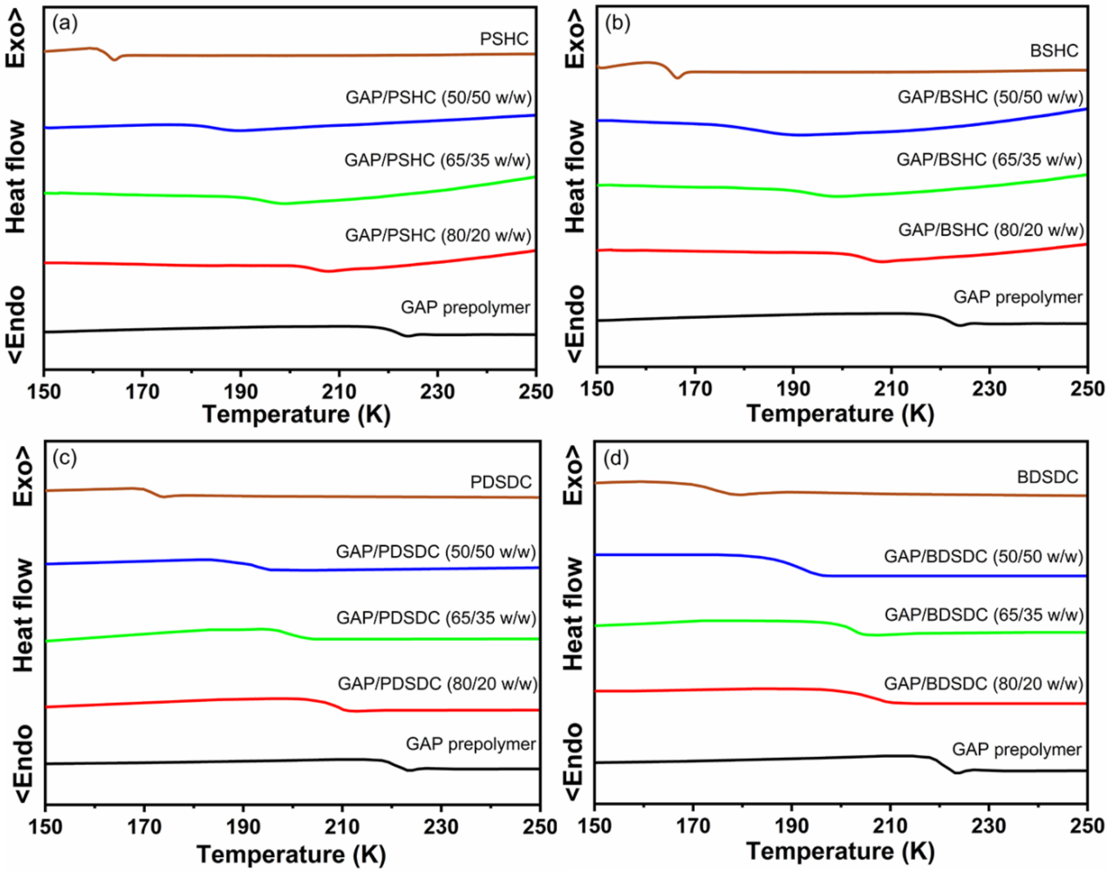

Fig. S9. DSC thermograms of (a) GAP/PSHC, (b) GAP/BSHC, (c) GAP/PDSDC, and (d) GAP/BDSDC.

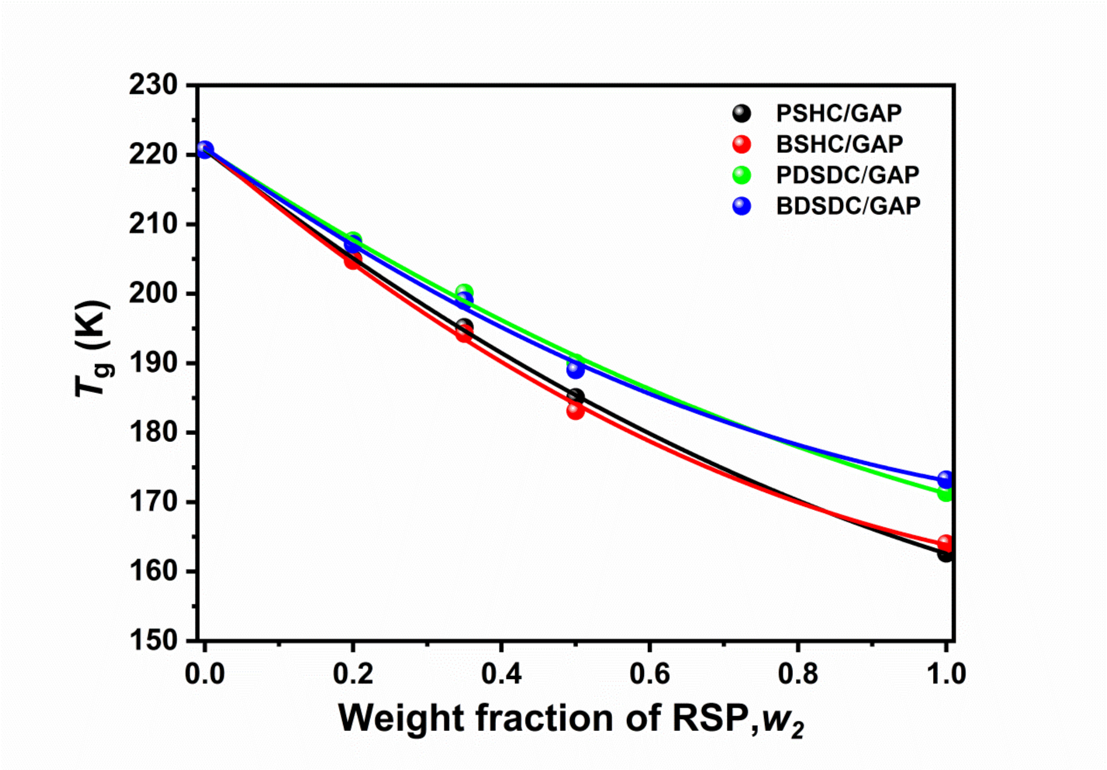

Fig. S10. Composition-dependent T_g of RGSs/GAP prepolymer binary mixtures.

Eq. S3 was applied successfully to the polymer-plasticizer binary mixtures to evaluate the miscibility between polymer and plasticizer [1].

$$I = \frac{T_g - T_{g1}\, w_1 - T_{g2}\, w_2}{w_1\, w_2} \qquad \text{(S3)}$$

where w_1 and w_2 are the weight fraction of the GAP prepolymer and RGS, respectively. T_{g1} and T_{g2} are T_g of the GAP prepolymer and RGS, respectively.

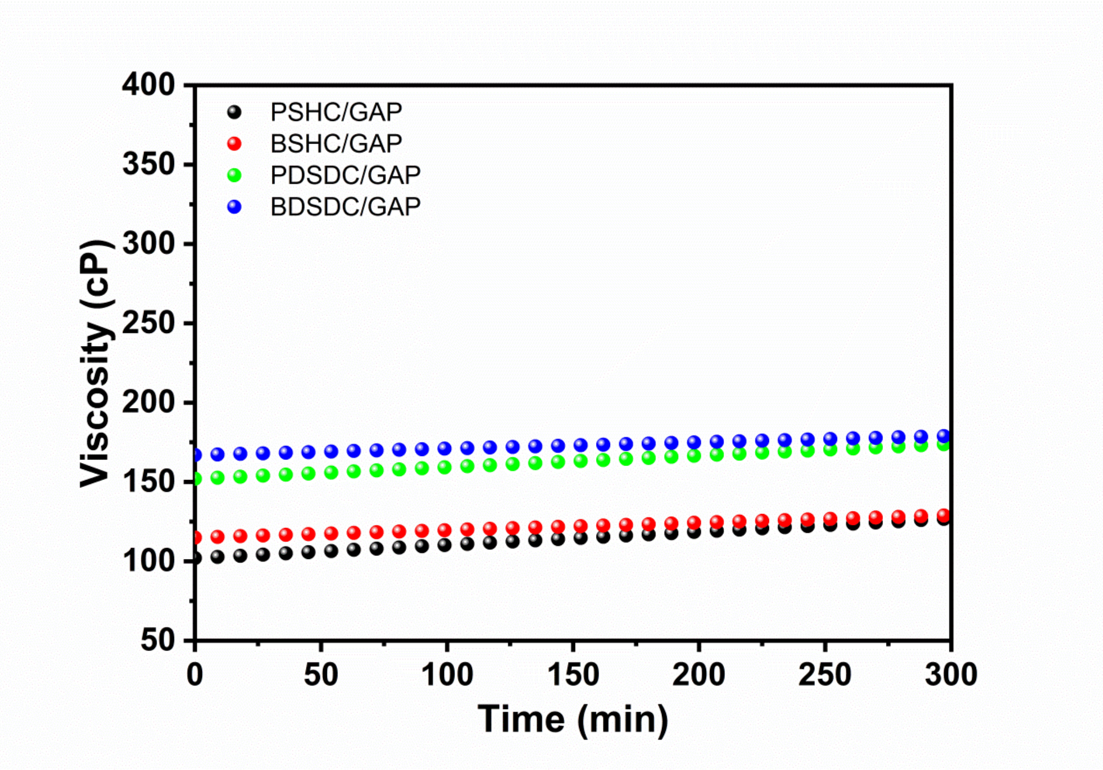

Fig. S11. Viscosity *versus* time for binary mixtures of RGSs/GAP prepolymer (50/50 w/w) isothermally at 30 ºC.

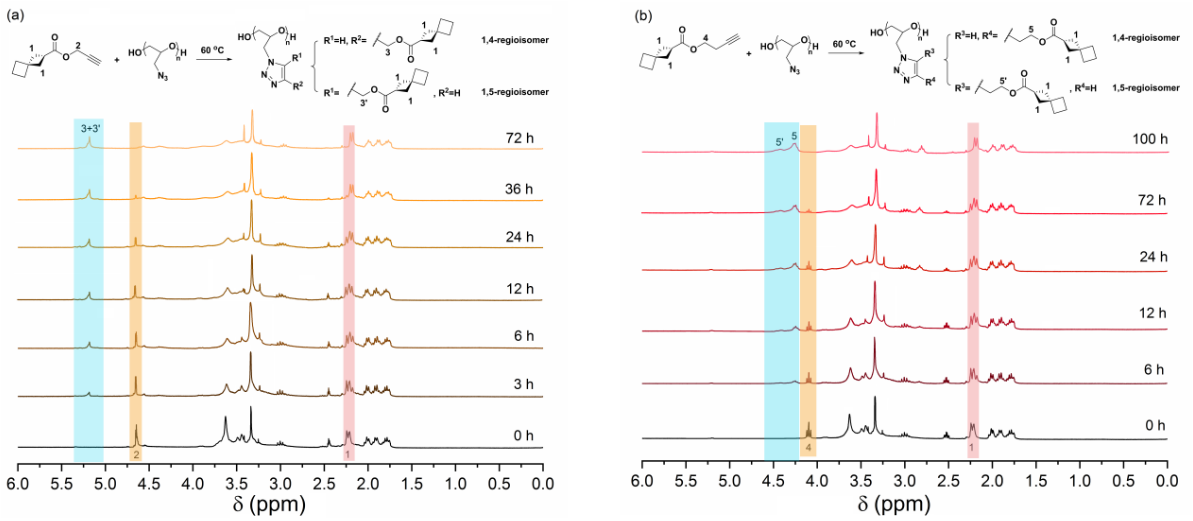

Fig. S12. Comparative ^{1}H NMR spectra of the catalyst-free azide-alkyne 1,3-DPCA reaction with respect to (a) PSHC (*n*=1) and (b) BSHC (*n*=2) toward the GAP prepolymer performed under the bulk conditions at 60 °C, respectively. The consumption of RSs could be traced more easily because of their more readily detectable resonance signals than by tracing the newly formed peaks of the triazole moiety.

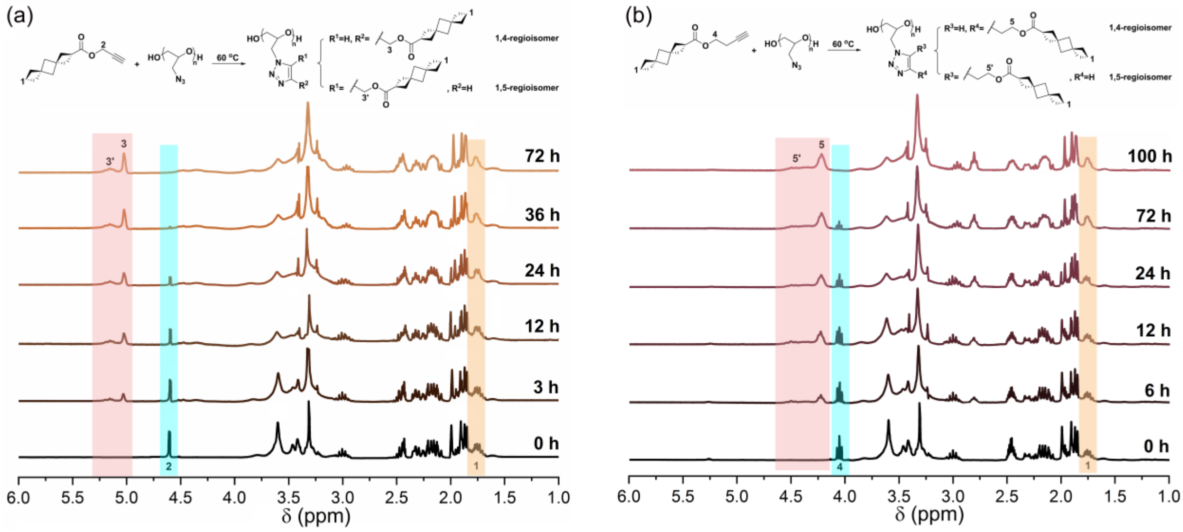

Fig. S13. Comparative ^{1}H NMR spectra of catalyst-free azide-alkyne 1,3-DPCA reaction with respect to (a) PDSDC (*n*=1) and (b) BDSDC (*n*=2) toward GAP prepolymer performed under the bulk condition at 60 °C, respectively.

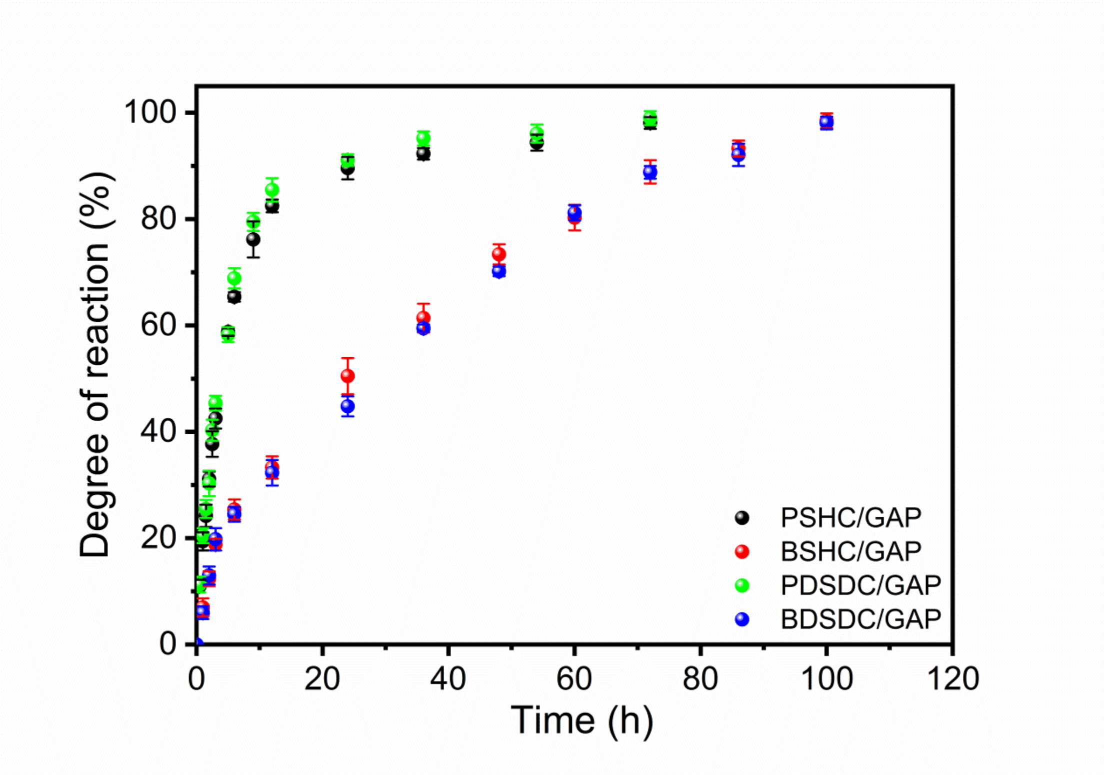

Fig. S14. Degree of reaction *versus* reaction time plot for the catalyst-free azide-alkyne 1,3-DPCA reaction of the RGSs with the GAP prepolymer at 60 ºC.

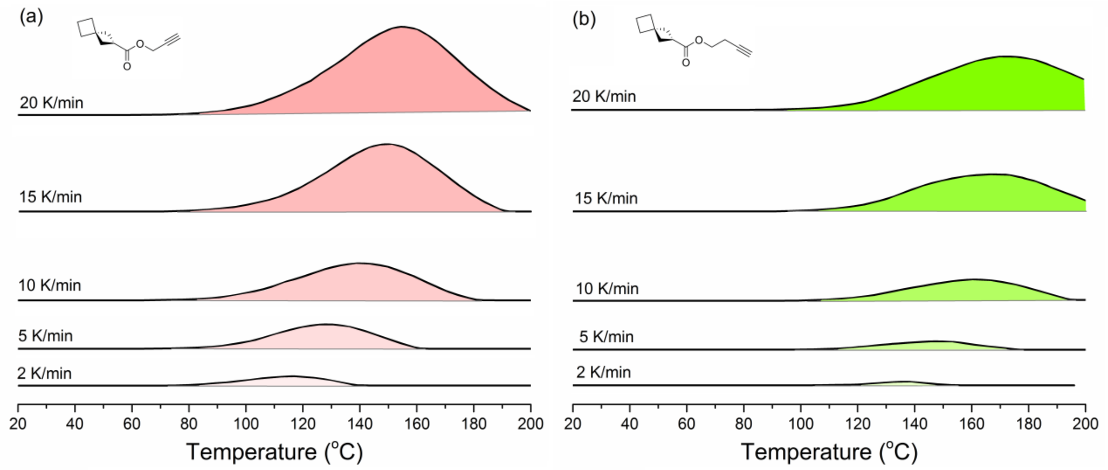

Fig. S15. Dynamic DSC curves of (a) PSHC (*n*=1)/GAP and (b) BSHC (*n*=2)/GAP mixtures at different heating rates.

The E_a of the click reaction was calculated from Eq. S4 based on ASTM E698 [2] along with combining Doyle's approximation [3].

$$\ln(\beta) = A - \frac{1.052\, E_a}{R\, T_{max}} \tag{S4}$$

where β is the scan rate (K min^{-1}), A is a constant, E_a is the activation energy (J mol^{-1}), T_{max} is the temperature at the maximum exothermic temperature (K) and R is the universal gas constant (8.314 J mol^{-1} K^{-1}) [4].

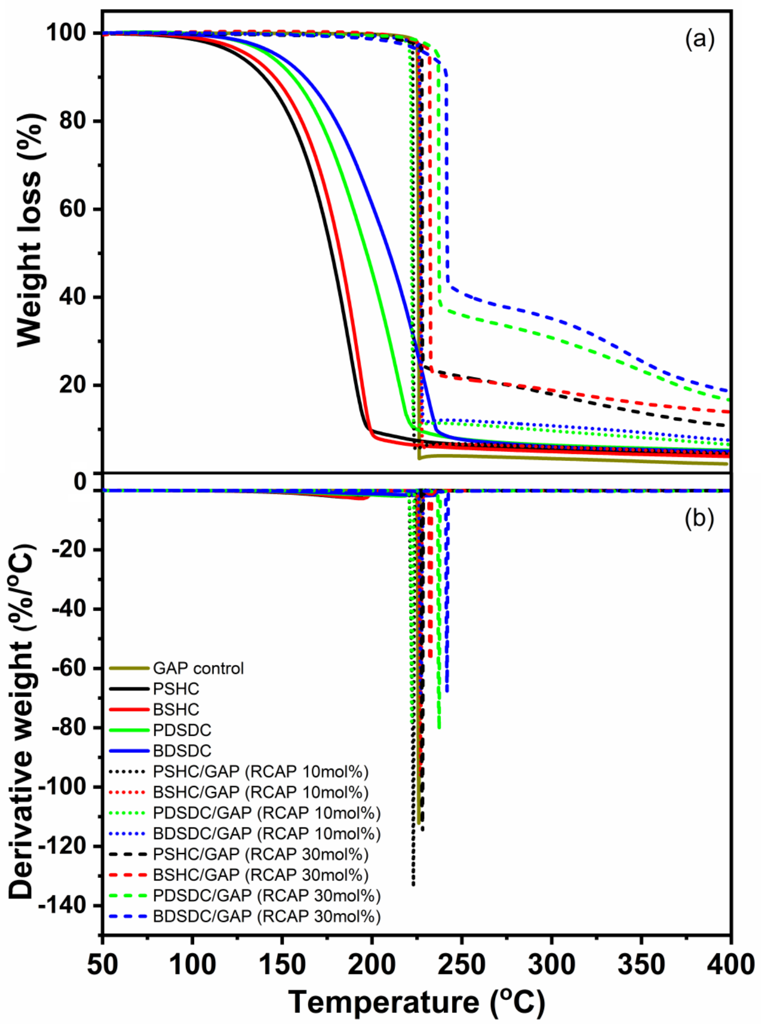

Fig. S16. (a) TGA and (b) DTG curves of the RGSs and RGSs@GAP-based PUs.

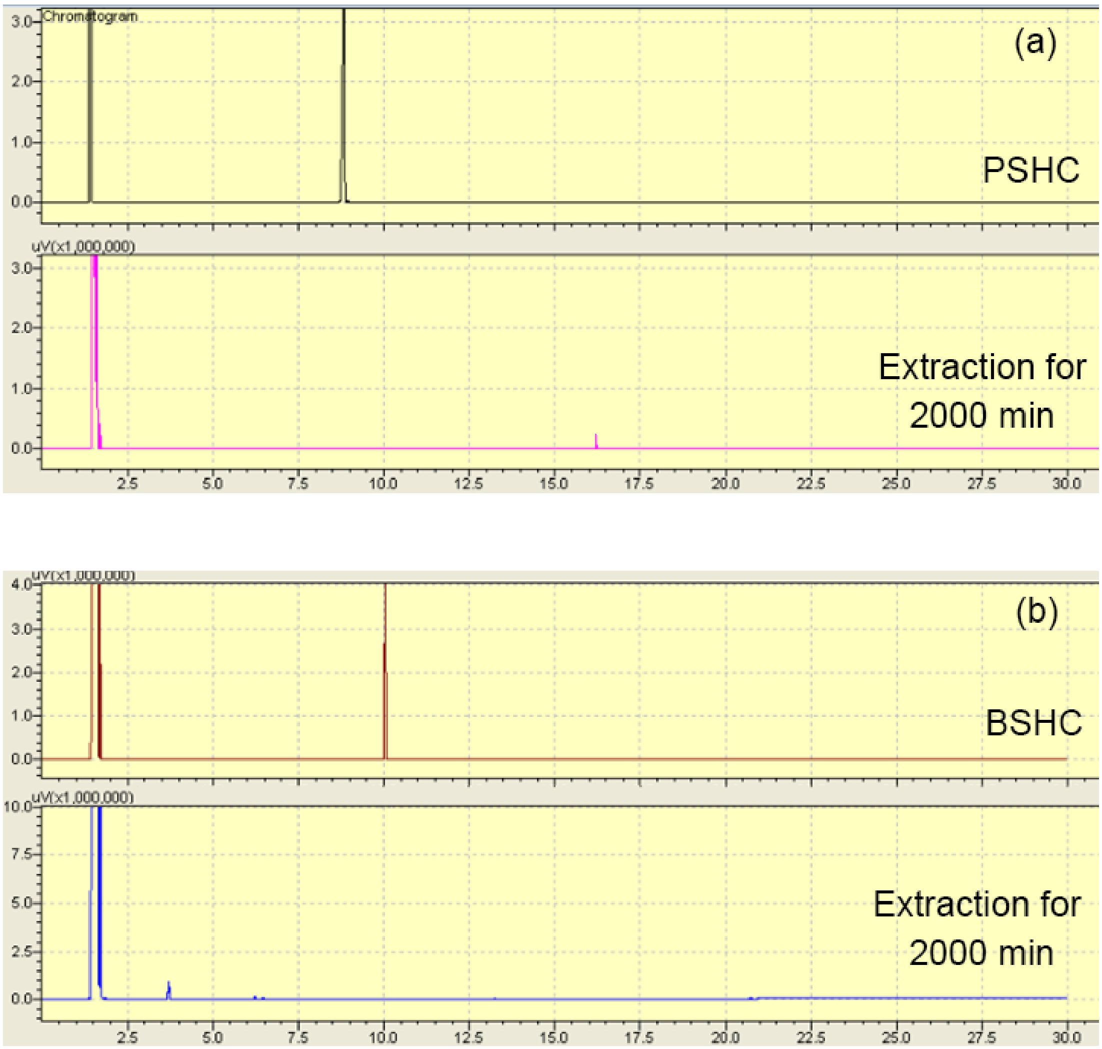

Fig. S17. GC spectra for detection of unreacted RGSs in (a) PSHC@GAP-based PU and (b) BSHC@GAP-based PU.

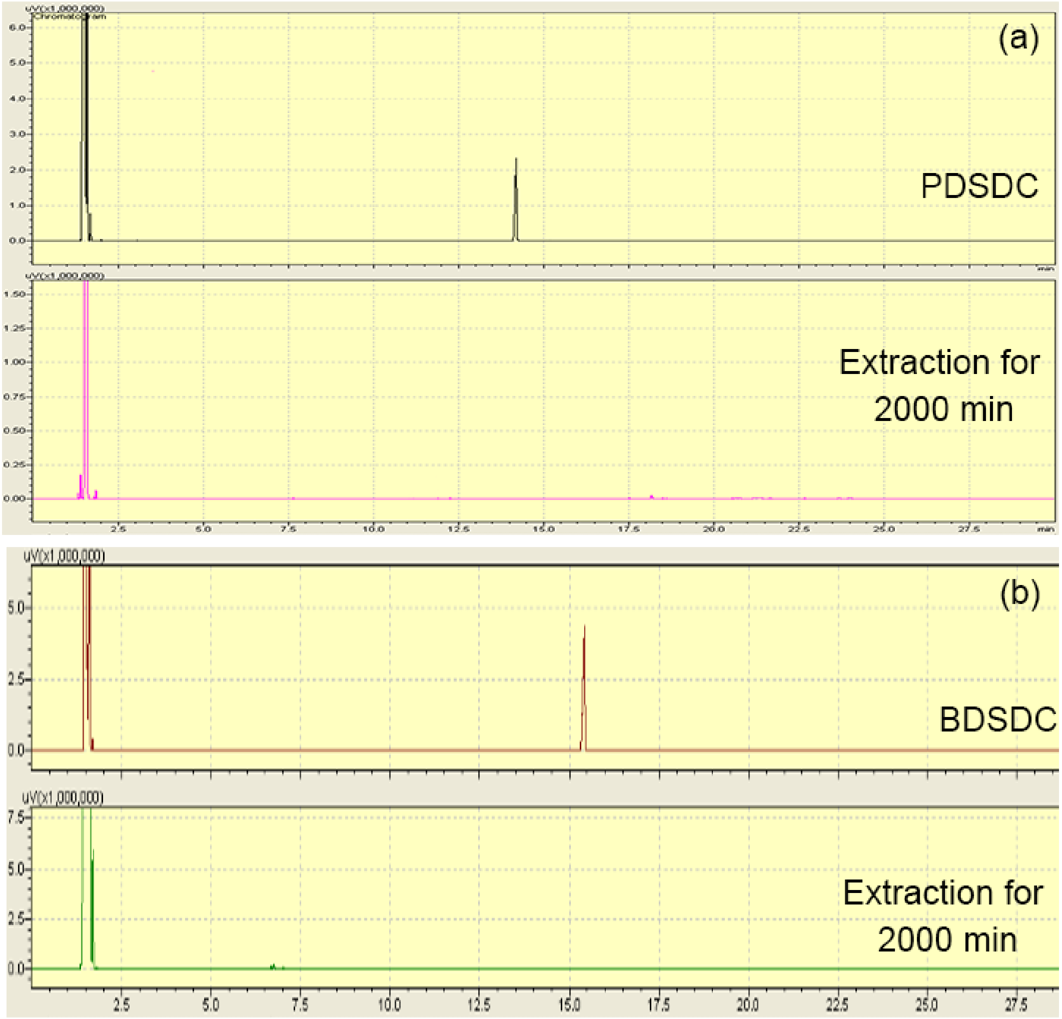

Fig. S18. GC spectra for detection of unreacted RGSs in (a) PDSDC@GAP-based PU and (b) BDSDC@GAP-based PU.

Table S1. LUMO and HOMO energy levels of RGS computed at the B3LYP/6-31G* level of theory.

RGS	Alpha occ. eigenvalues	Alpha virt. eigenvalues				
PSHC	-0.26519	0.00381	0.02481	0.04944	0.08438	0.09828
BSHC	-0.26586	0.00640	0.04758	0.05499	0.08434	0.09722
PDSDC	-0.25970	0.00391	0.02496	0.04958	0.08344	0.09113
BDSDC	-0.25997	0.00648	0.04769	0.05509	0.08327	0.09120

Table S2. Percentage of carbon (C), hydrogen (H), oxygen (O) and nitrogen (N), and empirical formula for the GAP control and RS/GAP-based PUs.

PU [a]	Elemental analysis (wt.%)				Experimental formula for 100 g
	C	H	O	N	
GAP control	38.5±0.7	5.3±0.9	17.1±0.4	39.1±0.8	$C_{3.21}H_{5.27}O_{1.07}N_{2.79}$
PSHC/GAP	49.5±0.6	6.1±0.7	17.1±0.7	27.3±0.5	$C_{4.12}H_{6.05}O_{1.07}N_{1.95}$
BSHC/GAP	50.3±0.4	6.3±0.7	16.8±0.6	26.6±0.8	$C_{4.19}H_{6.25}O_{1.05}N_{1.90}$
PDSDC/GAP	52.4±0.8	6.4±0.5	15.9±0.7	25.3±0.9	$C_{4.36}H_{6.32}O_{0.99}N_{1.81}$
BDSDC/GAP	53.2±0.3	6.6±0.5	15.5±0.8	24.7±0.4	$C_{4.43}H_{6.51}O_{0.97}N_{1.76}$

[a] Molar ratio of [C≡C]/[N_3]= 0.3/1.

$\Delta_f H^o$ can be calculated from the $\Delta_c H^o$ value based on the following equation,

$$\Delta_f H^o\,(C_aH_bO_cN_d,\ s) = a\,\Delta_f H^o\,(CO_2,\ g) + \frac{b}{2}\,\Delta_f H^o\,(H_2O,\ l) - \Delta_c H^o\,(C_aH_bO_cN_d,\ s) \qquad \text{(S5)}$$

where $\Delta_f H^o\,(CO_2,\ g) = -393.5$ kJ mol^{-1} and $\Delta_f H^o\,(H_2O,\ l) = -285.8$ kJ mol^{-1} [5].

References

[1] Wingborg, N.; Eldsäter, C.: 2,2-Dinitro-1,3-Bis-Nitrooxy-Propane (NPN): A New Energetic Plasticizer. Propellants, Explosives, Pyrotechnics, **27**, 314–319 (2002). .

[2] *Standard Test Method for Arrhenius Kinetic Constant for Thermally Unstable Materials (ANSI/ASTM E698-79)*; ASTM, Philadelphia, PA, USA;

[3] C. D. Doyle: Estimating isothermal life from thermogravimetric data. Journal of Applied Polymer Science, **6**, 639–642 (1962).

[4] Gorman, I. E.; Willer, R. L.; Kemp, L. K.; Storey, R. F.: Development of a triazole-cure resin system for composites: Evaluation of alkyne curatives. Polymer, **53**, 2548–2558 (2012).

[5] Gibbs, T. R.; Popolato, A.: *LASL Explosive Property Data*; University of California Press: Berkeley, CA; ISBN 0520040104.
